# Association of Early Adulthood 25-Year Blood Pressure Trajectories With Cerebral Lesions and Brain Structure in Midlife

**DOI:** 10.1001/jamanetworkopen.2022.1175

**Published:** 2022-03-10

**Authors:** Yi-Han Hu, Michael R. Halstead, R. Nick Bryan, Pamela J. Schreiner, David R. Jacobs, Stephen Sidney, Cora E. Lewis, Lenore J. Launer

**Affiliations:** 1 Laboratory of Epidemiology and Population Sciences, National Institute on Aging, Baltimore, Maryland; 2 Division of Neurocritical Care, Sentara Pulmonary, Critical Care, and Sleep Specialists, Norfolk, Virginia; 3 Department of Radiology, University of Pennsylvania, Philadelphia; 4 Division of Epidemiology and Community Health, University of Minnesota, Minneapolis; 5 Kaiser Permanente Medical Center Program, Oakland, California; 6 Department of Epidemiology, University of Alabama at Birmingham, Birmingham

## Abstract

**Importance:**

Midlife elevated blood pressure (BP) is an important risk factor associated with brain structure and function. Little is known about trajectories of BP that modulate this risk.

**Objective:**

To identify BP trajectory patterns from young adulthood to midlife that are associated with brain structure in midlife.

**Design, Setting, and Participants:**

This cohort study used data of US adults from Coronary Artery Risk Development in Young Adults (CARDIA), a prospective longitudinal study of Black and White men and women (baseline age 18 to 30 years) examined up to 8 times over 30 years (1985-1986 to 2015-2016). There were 885 participants who underwent brain magnetic resonance imaging (MRI) in the 25th or 30th year examinations. Analyses were conducted November 2019 to December 2020.

**Exposures:**

Using group-based trajectory modeling, 5 25-year BP trajectories for 3 BP traits were identified in the total CARDIA cohort of participants with 3 or more BP measures, which were then applied to analyses of the subset of 853 participants in the Brain MRI substudy. Mean arterial pressure (MAP) was examined as an integrative measure of systolic and diastolic BP. With linear regression, the associations of the BP trajectories with brain structures were examined, adjusting sequentially for demographics, cardiovascular risk factors, and antihypertensive medication use.

**Main Outcomes and Measures:**

Brain MRI outcomes include total brain, total gray matter, normal-looking and abnormal white matter volumes, gray matter cerebral blood flow, and white matter fractional anisotropy.

**Results:**

Brain MRI analyses were conducted on 853 participants (mean [SD] age, 50.3 [3.6] years; 399 [46.8%] men; 354 [41.5%] Black and 499 [58.5%] White individuals). The MAP trajectory distribution was 187 individuals (21.1%) with low-stable, 385 (43.5%) with moderate-gradual, 71 (8.0%) with moderate-increasing, 204 (23.1%) with elevated-stable, and 38 (4.3%) with elevated-increasing. Compared with the MAP low-stable trajectory group, individuals in the moderate-increasing and elevated-increasing groups were more likely to have higher abnormal white matter volume (moderate: β, 0.52; 95% CI, 0.23 to 0.82; elevated: β, 0.57; 95% CI, 0.19 to 0.95). Those in the MAP elevated-increasing group had lower gray matter cerebral blood flow (β, –0.42; 95% CI, –0.79 to –0.05) after adjusting for sociodemographics and cardiovascular risk factors. After adjustment for antihypertensive medication use, the difference was consistent for abnormal white matter volume, but results were no longer significant for gray matter cerebral blood flow.

**Conclusions and Relevance:**

Among young adults with moderate to high levels of BP, a gradual increase in BP to middle-age may increase the risk in diffuse small vessel disease and lower brain perfusion.

## Key Points

**Question:**

Are blood pressure trajectories in early adulthood associated with brain structure and integrity in midlife?

**Findings:**

In this cohort study of 853 adults aged 18 to 30 years who were followed up for 30 years, trajectories with a higher level of, or a gradual increase in, mean arterial pressure during early adulthood were associated with reduced brain health in midlife after adjusting for sociodemographics and cardiovascular risk factors.

**Meaning:**

These results suggest that, starting in early adulthood, the longitudinal pattern in blood pressure levels may be an indicator of increased risk for future poor brain health; preventing blood pressure increases as early as young adulthood may be warranted.

## Teaser

This cohort study of US adults using data from a longitudinal coronary study examines the association of trajectories of mean arterial pressure with brain health over 30 years of follow-up examination.

## Introduction

Clinical decisions about when to start treating elevated blood pressure (BP) are currently based on reducing the 10-year risk for atherosclerotic cardiovascular disease, including stroke. A recent randomized trial, the Systolic Blood Pressure Intervention Trial-Memory and Cognition in Decreased Hypertension,^1^ showed that treating systolic BP intensively to a goal of below 120 mm Hg compared with treating to the standard goal of below 140 mm Hg reduced a combined outcome of probable dementia and mild cognitive impairment. As is common, the history of an individual’s BP trajectory to the point of trial entry was not known. However, studies suggest knowing this history may improve clinical interpretations and decisions regarding treatment in mid- and late-life. Timely treatment of high BP could prevent known effects of elevated BP as a risk factor for cognitive impairment^2,3^ and dementia.^2,4^ Additionally, evidence suggests that midlife measures may be more informative than concurrently measured BP about future risk for preventing late-life cognitive impairment,^5,6^ dementia,^7,8^ and brain pathology.^9,10^

However, prior investigations on life-course BP and brain measures have predominantly focused on midlife to late-life BP patterns and subsequent outcomes.^7,8,9,10^ Typically, extant studies are based on only 1 measure of mid- and/or late-life BP, but there is evidence that pathology and cognitive disorders of late-life begin earlier than midlife,^11^ and there is evidence from cardiovascular disease studies that the trajectory of BP from youth to middle-age can modify the risk for cardiovascular diseases.^12^ Examining the association of young adulthood to midlife BP trajectories with brain integrity in midlife may provide insight into windows of opportunity for intervention and point to subgroups that may be at higher risk for later cognitive impairment.

To this end, we examined the associations of early adulthood systolic BP (SBP), diastolic BP (DBP), and mean arterial pressure (MAP) trajectories with brain magnetic resonance imaging (MRI) outcomes in midlife. We focused our main analyses on MAP because it has been found to have associations with memory function and brain health,^13,14^ and allows us to consider the integrated effects of both SBP and DBP on the brain.^9,15^ We hypothesize that BP trajectories with sustained elevated levels or gradual increases in BP over early adulthood are associated with increased risk of cerebral pathology as measured by brain atrophy, white matter lesion volume, and cerebral perfusion.

## Methods

### Study Design

The previously described Coronary Artery Risk Development in Young Adults (CARDIA) is a multicenter, prospective, longitudinal cohort study of 5115 healthy White and Black adults from 4 US metropolitan populations (Birmingham, Alabama; Chicago, Illinois; Minneapolis, Minnesota; Oakland, California).^16^ Participants were aged 18 to 30 years at baseline in 1985-1986 (year 0). Since enrollment, 8 follow-up examinations have been completed in years 2, 5, 7, 10, 15, 20, 25, and most recently in year 30 after baseline. As part of the ongoing cohort study, a subset of participants from 3 centers took part in the brain MRI substudy at year 25, year 30, or both (eMethods in the Supplement).^11^

Separate ethics approval was given by the institutional review boards from each field center and the coordinating center (University of Alabama Birmingham, University of Minnesota, and Kaiser Permanente Northern California), the MRI Reading Center (University of Pennsylvania), and the National Institutes of Health Office of Human Subjects Research Protection for the Intramural Research Program, National Institute on Aging. All participants provided written informed consent at each CARDIA examination, with a separate written consent for participation in the brain MRI substudy. This study followed the Strengthening the Reporting of Observational Studies in Epidemiology (STROBE) reporting guideline.

### Blood Pressure Measurements

BP was measured at each examination by trained technicians. After a 5-minute rest, SBP and DBP were measured 3 times at 1-minute intervals. A Hawksley random-zero sphygmomanometer was used for measurements from year 0 to year 20; and a digital sphygmomanometer (Omron Healthcare Inc) was used in the year 25 examination. The digital measurements were calibrated to random-zero sphygmomanometer values to avoid bias.^17^ SBP and DBP measurements used in the analysis were the average of second and third measurements. Three BP trajectories were examined in this study: SBP, DBP, and MAP, which was defined as the sum of one-third the SBP plus two-thirds of DBP.

### Brain MRI Data

Brain MRI was obtained in a subset of CARDIA participants in 3 research centers (Oakland, Minneapolis, and Birmingham) at year 25 (719 participants), year 30 (663 participants), or at both examinations (488 participants). All brain MRI scans were performed per CARDIA protocol using 3-Tesla magnetic resonance scanners, and standardized across machines using a common machine head phantom.^11^ Details on the MRI hardware and quality control are found in eMethods the Supplement.

MR images were processed using previously described methods^18,19,20,21^ to estimate brain volume measures (in cubic centimeters) as follows: total intracranial volume, total brain volume, total gray matter volume, total normal and abnormal white matter volume, and gray matter cerebral blood flow (a mean voxel-based blood flow in mL/100 g/min based on the arterial spin labeling data).^22^ The white matter fractional anisotropy was derived from diffusion tensor images as an indicator of microstructural tissue integrity, with values ranging from a zero (indicating unrestricted diffusion of water along the fiber tracts) to 1 (indicating the diffusion occurs along 1 axis, in all directions).

In the total sample, changes in MRI outcomes between year 25 and year 30 were small. Therefore, to increase our sample size we pooled the data from the 2 examinations. Of the 885 unique participants with MRI scans, 485 had repeated examinations of good quality. To account for this, per sequence, we calculated the weighted average for each brain measure. Weights were based on variance ratios of the year 25 and year 30 data (eMethods in the Supplement). The skewed distribution of abnormal white matter volume was log-transformed. All brain measures were transformed into *z* scores so that brain-related results could be compared across sequences on a standardized 1-SD increment.

### Covariates

Possible confounders of the association between BP and brain measures were identified from previous studies.^10,11^ We used covariate measures collected at the year 25 examination. For participants with year 30 MRI scans only, missing covariates from year 25 were replaced with year 30 values. We controlled for demographic strata used for the baseline sampling frame, including age, sex, self-identified Black or White race, and highest education level obtained; current smoking status (yes or no), current alcohol use (yes or no), and current antihypertensive medication use (yes or no) were assessed through self-report on questionnaires. Body mass index (BMI) was calculated as weight in kilograms divided by squared height in meters. A total physical activity score was calculated based on the Physical Activity Questionnaire.^23^ Diabetes was defined by the presence of 1 of the following: diagnosis of diabetes, history of hypoglycemic medication use, fasting glucose levels above 126 mg/dL (to convert to millimoles per liter, multiply by 0.0555), 2-hour postload glucose tolerance test above 200 mg/dL, or hemoglobin A_1c_ levels 6.5% or greater (to convert to proportion of total hemoglobin, multiply by 0.01).

### Cognitive Function

Cognitive impairment has been associated with our brain outcomes of interest.^5,6^ As an exploratory analysis, we examined the associations of BP trajectories with 3 different cognitive function tests measured at year 30, including the Digital Symbol Substitution Test (DSST), the Rey Auditory Verbal Learning Test (RAVLT; delayed [25 minutes] free recall score was used), and the Stroop Interference Test (the interference score was used) (eTable 6 in the Supplement).

### Analytical Sample

We modeled the BP trajectories among 4677 participants who had at least 3 repeated BP measures from year 0 to year 25 (hereafter referred to as overall sample); 853 with complete MRI and BP measures were included in the analyses (eFigure in the Supplement). We note that, compared with those not included in the MRI study, participants in the MRI study were more often Black, had lower mean BMI, and were less likely to have prevalent hypertension or take antihypertensive medication (eTable 1 in the Supplement). Additionally, fewer MRI participants were assigned to moderate-increasing trajectory and elevated-increasing trajectory groups when compared with the trajectory assignment in the overall sample. Of the participants with BP trajectory assignments, the 2736 who had completed 3 cognitive function tests at the year 30 examination were used for an exploratory analysis of the associations of BP trajectories and cognitive function outcomes.

### Statistical Analysis

Trajectories were estimated with group-based trajectory modeling SAS Proc Traj version 9.4. Group-based trajectory modeling is a semi-parametric procedure designed to identify subgroups with similar changes over time or age.^24,25,26,27,28^ The detailed procedure has been described previously (eMethods in the Supplement).^12^

To investigate the association of BP-specific trajectory groups with brain outcomes at years 25 and/or 30, we estimated several multivariable-adjusted regression models: model 1 included BP trajectory groups, demographic factors (age, sex, race, educational attainment) and field center; model 2 additionally adjusted for cardiovascular risk factors (including current smoking status, current alcohol use, BMI, physical activity level, and diabetes status); model 3 additionally adjusted for antihypertensive medication, as use of antihypertensives could change the BP trajectories in a manner that would provide us with additional information about the effects of treatment on our findings. The models for volumetric outcomes (ie, total brain volume, total gray matter volume, total normal white matter volume, and abnormal white matter volume) were adjusted for total intracranial volume, while the models for gray matter cerebral blood flow and white matter fractional anisotropy were adjusted for total brain volume. The trajectory with the lowest group mean of BP was used as the reference trajectory (ie, the low-stable group in the Figure).

**Figure.**
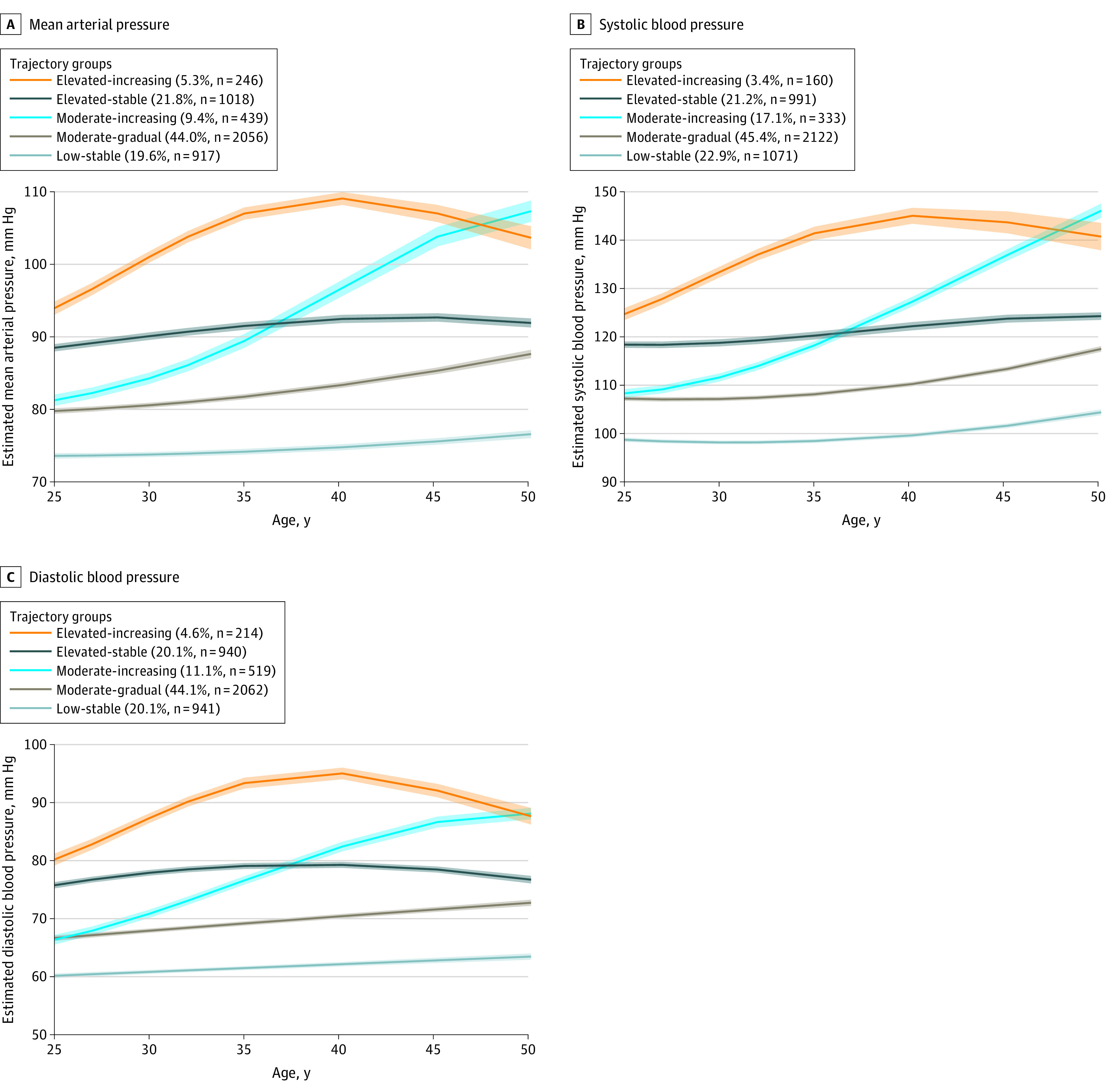
Trajectory of Estimated Blood Pressure in the Coronary Artery Risk Development in Young Adults Study Shaded areas indicate 95% CIs.

Our primary trait of interest was MAP, as it provides an integrated measure of SBP and DBP, both of which have been shown to have associations with small vessel disease and dementia.^7,9^ Secondary analyses examined the associations of SBP and DBP with the brain outcomes of interest.

Results were adjusted for multiple comparisons using the Benjamini-Hochberg method for false discovery rate^29^; statistical significance was set to false discovery rate–adjusted *P* value (ie, q-value) of 0.10 or less in 2-sided tests. Data analysis was conducted in R version 3.6.3 (R Project for Statistical Computing) and SAS version 9.4 (SAS Institute Inc).

## Results

### Blood Pressure Trajectories and Study Sample Characteristics

Of the 885 participants of the brain MRI substudy, age ranged between 42 and 61 years (mean [SD] age, 50.3 [3.6] years); participants comprised 419 (47.3%) men, and 366 (41.4%) Black and 499 (58.5%) White Americans (Table 1). BP measures and hypertension prevalence at each examination for each trajectory are reported in eTables 2 and 3 in the Supplement.

**Table 1.** Descriptive Statistics in the Magnetic Resonance Imaging Subset by MAP Trajectory Group in the Coronary Artery Risk Development in Young Adults Cohort Year 25 and Year 30 Examination^a^

Characteristics	Participants, No. (%)					
	Overall (n = 885)	Low-stable (n = 187)	Moderate-gradual (n = 385)	Moderate-increasing (n = 71)	Elevated-stable (n = 204)	Elevated-increasing (n = 38)
**Demographic characteristics**						
Total examinations, mean (SD), No.^b^	7.7 (0.8)	7.7 (0.7)	7.7 (0.8)	7.6 (0.8)	7.6 (0.8)	7.6 (0.7)
Age, mean (SD), y	50.3 (3.6)	50.6 (3.6)	50.4 (3.6)	49.8 (3.7)	50.2 (3.6)	49.8 (3.6)
Sex						
Men	419 (47.3)	38 (20.3)	169 (43.9)	31 (43.7)	153 (75.0)	28 (73.7)
Women	466 (52.7)	149 (79.7)	216 (56.1)	40 (56.3)	51 (25.0)	10 (26.3)
Race						
Black	366 (41.4)	26 (13.9)	163 (42.3)	49 (69.0)	101 (49.5)	27 (71.1)
White	519 (58.6)	161 (86.1)	222 (57.7)	22 (31.0)	103 (50.5)	11 (28.9)
Highest education						
High school or less	349 (39.4)	51 (27.2)	148 (38.4)	34 (47.9)	94 (46.1)	22 (57.9)
College	400 (45.2)	97 (51.9)	181 (47.0)	26 (36.6)	82 (40.2)	14 (36.8)
Graduate school	136 (15.4)	39 (20.9)	56 (14.6)	11 (15.5)	28 (13.7)	2 (5.3)
**MAP, mean (SD), mm Hg^c^**						
Mean from year 0 to year 25	84.2 (7.6)	74.2 (3.0)	82.6 (2.8)	90.9 (5.1)	90.8 (2.9)	101.3 (3.8)
Year 0 exam	82.7 (8.6)	75.5 (6.3)	81.3 (6.3)	82.9 (7.5)	89.5 (6.5)	96.7 (8.8)
Year 25 exam	88.1 (11.8)	75.6 (6.5)	87.5 (7.8)	105.9 (10.5)	91.2 (7.8)	105.4 (13.2)
**Clinical measures**						
Hypertension^d^	437 (49.4)	12 (6.4)	158 (41.0)	70 (98.6)	159 (77.9)	38 (100.0)
Taking antihypertensive medication^e^	211 (23.9)	7 (3.7)	58 (15.2)	36 (50.7)	82 (40.2)	28 (73.7)
BMI						
<25	253 (28.6)	92 (49.2)	112 (29.1)	7 (9.9)	36 (17.7)	6 (15.8)
≥25 to <30	310 (35.0)	65 (34.8)	135 (35.1)	24 (33.8)	68 (33.3)	18 (47.4)
≥30	322 (36.4)	30 (16.0)	138 (35.8)	40 (56.3)	100 (49.0)	14 (36.8)
Diabetes^f^	116 (13.1)	14 (7.5)	39 (10.1)	20 (28.2)	35 (17.2)	8 (21.1)
**Behavior**						
Current smoker^g^	148 (16.9)	22 (11.9)	63 (16.4)	21 (30.4)	33 (16.3)	9 (24.3)
Alcohol use	692 (78.2)	156 (83.4)	293 (76.1)	53 (74.6)	163 (79.9)	27 (71.1)
Physical activity, median (IQR), intensity units^h^	291.0 (144.0-507.0)	312.0 (171.5-499.0)	297.0 (149.0-516.0)	288.0 (108.0-501.0)	275.0 (138.0-512.3)	170.5 (48.0-402.0)

Abbreviations: BMI, body mass index (calculated as weight in kilograms divided by height in meters squared); DBP, diastolic blood pressure; MAP, mean arterial pressure; SBP, systolic blood pressure.

^a^ All data from the Coronary Artery Risk Development in Young Adults study, 1985-2016. All covariates (ie, sociodemographics, clinical measures, and behavior) were collected at the year 25 examination. For participants with year 30 MRI only, missing covariates were updated using year 30 information.

^b^ Number of visits ranged from 3 to 8.

^c^ MAP calculated by adding one-third of SBP and two-thirds of DBP.

^d^ SBP 130 mm Hg or higher or DBP 80 mm Hg or higher or taking antihypertensive medication.

^e^ Three participants without antihypertensive medication use status.

^f^ One participant without diabetes diagnosis.

^g^ Seven participants without smoking status.

^h^ Total activity intensity in the past year.

Five trajectory groups for each of MAP, SBP, and DBP were estimated and labeled as follows: (1) low-stable group, consisting of participants who maintained low BP levels throughout the study period (MAP, 917 individuals [19.6%]; SBP, 1071 individuals [22.9%]; DBP, 941 individuals [20.1%]); (2) moderate-gradual group, comprising individuals who started at moderate BP levels and experienced gradual increases (MAP, 2056 individuals [44.0%]; SBP, 2122 individuals [45.4%]; DBP, 2062 individuals [44.1%]); (3) moderate-increasing group, who started at moderate levels and experienced a more rapid increase (MAP, 439 individuals [9.4%]; SBP, 333 individuals [17.1%]; DBP, 519 individuals [11.1%]); (4) elevated-stable group, who started at a relatively higher BP level that was stable throughout the follow-up period (MAP, 1018 individuals [21.8%]; SBP, 991 individuals [21.2%]; DBP, 940 individuals [20.1%]); and, (5) elevated-increasing group, who started at a relatively elevated level, gradually increased until age 40 years, and then moderately decreased (MAP, 246 individuals [5.3%]; SBP, 160 individuals [3.4%]; DBP, 214 individuals [4.6%]) (Figure). Similar trajectory groups were identified for SBP and DBP (eTable 1 in the Supplement), while fewer participants were assigned to the SBP moderate-increasing and elevated-increasing groups compared with the corresponding DBP or MAP trajectory groups.

### Blood Pressure Trajectory and Brain Outcomes

The descriptive statistics of brain outcomes showed that the MAP elevated-increasing group had the smallest total gray matter volume to intracranial volume ratio (mean [SD] ratio: 0.46 [0.03] vs low-stable, 0.47 [0.02]; *P* = .06), highest abnormal white matter volume (0.25 [0.20]vs low-stable, 0.18 [0.11]; *P* < .001), and lowest gray matter cerebral blood flow (mean [SD]: 26.9 [5.4] mL/100 g/min vs low-stable, 30.7 [7.6] mL/100 g/min) across all 5 groups (Table 2). A similar pattern was found for the SBP and DBP trajectory groups (eTable 4 in the Supplement).

**Table 2.** Brain Characteristics of the Magnetic Resonance Imaging Subset by Mean Arterial Pressure Trajectory Group in the Coronary Artery Risk Development in Young Adults Cohort Year 25 and Year 30 Examination^a^

Characteristics	Participants, No. (%)						*P* value^b^
	Overall	Low-stable	Moderate-gradual	Moderate-increasing	Elevated-stable	Elevated-increasing	
Total brain volume							
Valid participants	853	183 (21.5)	371 (43.5)	67 (7.9)	198 (23.2)	34 (3.9)	NA
Mean (SD), cm^3^^c^	0.85 (0.03)	0.85 (0.03)	0.85 (0.03)	0.86 (0.03)	0.85 (0.03)	0.85 (0.04)	.12
Gray matter volume							
Valid participants	853	183 (21.5)	371 (43.5)	67 (7.9)	198 (23.2)	34 (3.9)	NA
Mean (SD), cm^3^^c^	0.46 (0.02)	0.47 (0.02)	0.47 (0.02)	0.46 (0.02)	0.46 (0.02)	0.46 (0.03)	.06
White matter volume							
Valid participants	846	182 (21.5)	369 (43.6)	66 (7.8)	195 (23.1)	34 (4.0)	NA
Normal-looking white matter, mean (SD), cm^3^^c^	0.38 (0.02)	0.38 (0.02)	0.38 (0.02)	0.39 (0.02)	0.38 (0.02)	0.38 (0.02)	.41
Abnormal white matter, mean (SD), cm^3^^d^	0.19 (0.14)	0.18 (0.11)	0.18 (0.13)	0.26 (0.24)	0.18 (0.13)	0.25 (0.20)	<.001
White matter fractional anisotropy							
Valid participants	777	168 (21.6)	341 (43.9)	58 (7.5)	181 (23.3)	29 (3.7)	NA
Mean (SD)	0.25 (0.02)	0.26 (0.02)	0.25 (0.02)	0.25 (0.02)	0.25 (0.02)	0.24 (0.02)	.003
Gray matter cerebral blood flow							
Valid participants	753	171 (22.7)	329 (43.7)	56 (7.4)	167 (22.2)	30 (4.0)	NA
Mean (SD), mL/100 g/min	29.72 (7.22)	30.71 (7.55)	30.38 (7.31)	30.17 (6.92)	27.79 (6.63)	26.90 (5.39)	<.001

Abbreviation: NA, not applicable.

^a^ All data from the Coronary Artery Risk Development in Young Adults study, 1985-2016, and based on the cases with complete data on the outcome and all covariates.

^b^ *P* value was based on 1-way analysis of variance.

^c^ Reported as brain volume to intracranial volume ratio.

^d^ Log transformation was applied due to skew distribution.

In a linear regression model adjusting for demographic variables (model 1 in Table 3), compared with the low-stable MAP group, the moderate-increasing group had lower white matter fractional anisotropy (β, –0.33 [95% CI, –0.62 to –0.04] vs low-stable mean, 0.24) and more abnormal white matter volume (β, 0.55 [95% CI, 0.26 to 0.84] vs low-stable mean, 0.23); the elevated-stable group had lower white matter fractional anisotropy (β, –0.29 [95% CI, –0.51 to –0.07] vs low-stable mean, 0.24) and gray matter cerebral blood flow (β, –0.24 [95% CI, –0.45 to –0.03] vs low-stable mean, 0.49), and the elevated-increasing group had smaller total gray matter volume (β, –0.18 [95% CI, –0.35 to –0.01] vs low-stable mean, –0.07), more abnormal white matter volume (β, 0.60 [95% CI, 0.22 to 0.97] vs low-stable mean, 0.23), and lower gray matter cerebral blood flow (β, –0.48 [95% CI, –0.85 to –0.12] vs low-stable mean, 0.49). In general, with the addition of cardiovascular risk factors, effect size was closer toward zero and not significant (model 2, Table 3). After additional adjustment for antihypertensive medication use, the results for gray matter cerebral blood flow and white matter fractional anisotropy were not significant (model 3, Table 3). There remained a significant difference between the moderate-increasing and low-stable groups in abnormal white matter volume after full adjustment (β, 0.48 [95% CI, 0.17 to 0.78] vs low-stable mean, 0.23).

**Table 3.** The Association of Mean Arterial Pressure Trajectory With Brain Outcomes vs Low-stable Reference Trajectory^a^

Estimation	Low-stable (n = 183)	Moderate-gradual (n = 371)		Moderate-increasing (n = 67)		Elevated-stable (n = 198)		Elevated-increasing (n = 34)	
	Mean (SE)^b^	β (95% CI)	*q* ^c^	β (95% CI)	*q* ^c^	β (95% CI)	*q* ^c^	β (95% CI)	*q* ^c^
**Model 1**									
Total									
Brain volume	–0.04 (0.03)	–0.003 (–0.06 to 0.05)	0.95	–0.01 (–0.10 to 0.08)	0.90	–0.03 (–0.10 to 0.04)	0.58	–0.11 (–0.23 to 0.002)	0.11
Gray matter volume	–0.07 (0.05)	0.01 (–0.07 to 0.09)	0.90	–0.03 (–0.16 to 0.09)	0.76	–0.003 (–0.10 to 0.10)	0.96	–0.18 (–0.35 to –0.01)	0.07
Normal white matter volume	–0.02 (0.04)	–0.02 (–0.08 to 0.05)	0.74	–0.01 (–0.11 to 0.09)	0.95	–0.06 (–0.14 to 0.02)	0.23	–0.04 (–0.17 to 0.09)	0.73
Abnormal white matter volume^d^	0.23 (0.10)	0.04 (–0.14 to 0.22)	0.82	0.55 (0.26 to 0.84)	0.003	0.10 (–0.11 to 0.32)	0.55	0.60 (0.22 to 0.97)	0.01
White matter fractional anisotropy	0.24 (0.11)	–0.09 (–0.27 to 0.09)	0.55	–0.33 (–0.62 to –0.04)	0.06	–0.29 (–0.51 to –0.07)	0.03	–0.33 (–0.72 to 0.06)	0.19
Gray matter cerebral blood flow	0.49 (0.10)	–0.02 (–0.19 to 0.15)	0.90	–0.11 (–0.40 to 0.17)	0.61	–0.24 (–0.45 to –0.03)	0.06	–0.48 (–0.85 to –0.12)	0.03
**Model 2**									
Total									
Brain volume	0.03 (0.04)	–0.002 (–0.06 to 0.05)	0.98	0.01 (–0.08 to 0.10)	0.95	–0.02 (–0.09 to 0.04)	0.75	–0.10 (–0.22 to 0.01)	0.17
Gray matter volume	0.003 (0.06)	0.01 (–0.07 to 0.10)	0.91	0.002 (–0.13 to 0.13)	0.98	0.003 (–0.10 to 0.10)	0.98	–0.18 (–0.34 to –0.01)	0.14
Normal white matter volume	0.04 (0.04)	–0.02 (–0.08 to 0.05)	0.87	0.003 (–0.10 to 0.11)	0.98	–0.06 (–0.13 to 0.02)	0.33	–0.03 (–0.16 to 0.11)	0.89
Abnormal white matter volume^d^	0.25 (0.13)	0.04 (–0.15 to 0.22)	0.89	0.52 (0.23 to 0.82)	0.009	0.11 (–0.12 to 0.33)	0.61	0.57 (0.19 to 0.95)	0.03
White matter fractional anisotropy	0.22 (0.13)	–0.07 (–0.25 to 0.11)	0.75	–0.28 (–0.58 to 0.02)	0.17	–0.27 (–0.49 to –0.05)	0.10	–0.25 (–0.64 to 0.13)	0.36
Gray matter cerebral blood flow	0.71 (0.12)	0.04 (–0.13 to 0.22)	0.89	–0.01 (–0.30 to 0.28)	0.98	–0.15 (–0.37 to 0.07)	0.33	–0.42 (–0.79 to –0.05)	0.10
**Model 3**									
Total									
Brain volume	0.03 (0.04)	0.004 (–0.05 to 0.06)	0.93	0.03 (–0.06 to 0.12)	0.88	–0.01 (–0.08 to 0.06)	0.91	–0.07 (–0.19 to 0.05)	0.55
Gray matter volume	0.01 (0.06)	0.02 (–0.06 to 0.10)	0.88	0.02 (–0.11 to 0.16)	0.89	0.02 (–0.08 to 0.13)	0.88	–0.14 (–0.32 to 0.04)	0.43
Normal white matter volume	0.04 (0.04)	–0.01 (–0.08 to 0.05)	0.88	0.02 (–0.09 to 0.13)	0.89	–0.04 (–0.12 to 0.04)	0.65	–0.00001 (–0.14 to 0.14)	1.00
Abnormal white matter volume^d^	0.23 (0.13)	0.02 (–0.16 to 0.21)	0.91	0.48 (0.17 to 0.78)	0.04	0.06 (–0.18 to 0.30)	0.88	0.49 (0.09 to 0.89)	0.20
White matter fractional anisotropy	0.24 (0.13)	–0.05 (–0.23 to 0.13)	0.88	–0.22 (–0.53 to 0.08)	0.46	–0.22 (–0.45 to 0.02)	0.37	–0.16 (–0.57 to 0.24)	0.78
Gray matter cerebral blood flow	0.70 (0.12)	0.04 (–0.14 to 0.21)	0.88	–0.02 (–0.32 to 0.27)	0.93	–0.16 (–0.39 to 0.07)	0.46	–0.44 (–0.83 to –0.05)	0.23

^a^ Total included participants: volume of total brain, gray matter, and hippocampus, 853 participants; normal-looking white matter and abnormal white matter volume, 846 participants; white matter fractional anisotropy and mean diffusivity, 777 participants; and cerebral blood flow, 753 participants. All outcomes were standardized to *z* score values. All models were adjusted for age, sex, race, education, and field center. In model 2, body mass index, diabetes, physical activity level (log-transformed), current smoking status, and alcohol use were added. Model 3 was additionally adjusted for antihypertensive medication. Tissue volume measures models additionally adjusted for total intracranial volume; white matter fractional anisotropy and gray matter cerebral blood flow models additionally adjusted for total brain volume.

^b^ Adjusted means based on the estimated model when intracranial volume/total brain volume was standardized, and age and physical activity level centered. Data drawn from the Coronary Artery Risk Development in Young Adults (CARDIA) study, 1985-2016.

^c^ *q* Value was calculated using Benjamini-Hochberg method for the adjustment of multiple comparison. A *q* ≤ 0.10 was considered statistically significant.

^d^ Abnormal white matter volume was log-transformed before standardizing to *z* score values.

The SBP trajectories in the moderate-increasing and elevated-increasing groups had higher abnormal white matter volume compared with the low-stable group in the full-adjustment model (eTable 5 in the Supplement). For DBP, adjusting for demographic variables, compared with the low-stable group, the elevated-increasing group had lower gray matter cerebral blood flow and more abnormal white matter volume. These results were not significant after adjustment of cardiovascular risk variables (eTable 5 in the Supplement). In our exploratory analysis of the association between cognitive impairment and brain function, the moderate-increasing, elevated-increasing, and elevated-stable groups scored relatively worse on executive function (Stroop inference test) and verbal memory (RAVLT) (eTable 6 in the Supplement).

## Discussion

The main finding of this study was that, compared with BP trajectories that capture low levels and stable trajectories from young adulthood to middle age, trajectories with a gradual increase in MAP (ie, moderate-increasing and elevated-increasing) were more likely to have indications of poor brain health, including lower total gray matter volume, abnormal white matter volume, and lower gray matter cerebral blood flow after adjusting for sociodemographic factors and multiple comparisons. After adjusting for cardiovascular risk factors, most of these associations were no longer significant, and all associations were not significant after adjustment for antihypertensive medication use except for abnormal white matter volume in the moderate-increasing group. We also found the elevated-stable MAP trajectory group had significantly lower white matter fractional anisotropy, but the difference with the low-stable group was not significant with the addition of antihypertension medication use. The moderate-increasing and elevated-increasing SBP trajectories associated with abnormal white matter volume remained significant after full adjustment. Together, this suggests less favorable MAP trajectories, due mainly to increasing SBP, are associated with more diffuse brain changes, which may be ameliorated with cardiovascular risk control, specifically BP control. An increase in MAP may signal physiologic or autoregulation changes that reduce the brain’s capacity to protect itself.

Midlife hypertension,^30^ higher SBP,^30,31^ DBP,^32,33^ and MAP^15^ have all been associated with the development of, or higher than usual levels of, abnormal white matter volume in late life. Mechanisms underlying the relationship of BP with these cerebral pathologies are under investigation. A leading hypothesis suggests elevated BP impacts the brain through changes in cerebrovascular autoregulation and damage to small or micro vessels.^34^ Whether or not trajectories of higher levels of the BP characteristics are informative about future risk has not been well-studied in young to middle age, which is when some people begin to experience gradually changes in BP level. Furthermore, the factor most associated with abnormal white matter volume progression and related cognitive impairment, besides age, is already having abnormal white matter volume, making it important to understand when and how abnormal white matter volume may be initiated.^35^ In a previous cross-sectional study based on the CARDIA year 25 MRI data,^11^ the authors found that elevations in SBP and DBP were associated with abnormal white matter volume, white matter fractional anisotropy, and gray matter cerebral blood flow. Our study suggests 2 groups in particular are at risk for having abnormal white matter volume, ie, those who started at baseline with moderate to high levels of BP and experienced gradual increases in MAP and in SBP. Of note, those who remained stable at higher BP levels (ie, the elevated-stable group) had similar abnormal white matter volume to those with lower BP at baseline (moderate-gradual group). Also, the elevated-stable group had an average MAP from year 0 to year 25 similar to that of the moderate-increasing group. These results suggest that, along with level, the rate of BP increase should be monitored, as even gradual increases may confer extra physiologic stress leading to additional brain pathology. The results also highlight the fact that, without historical data on the individual level, baseline groupings lump together individuals with different prospective risks. Furthermore, the association of SBP moderate-gradual or elevated-gradual groups with abnormal white matter volume remained significant after adjusting for last BP measure (eTable 7 in the Supplement), suggesting knowledge of the long-term trajectory adds additional information on brain health than 1 cross-sectional measure of BP level.

In an exploratory analysis, compared with the MAP low-stable group, besides moderate-increasing and elevated-increasing groups, those in the elevated-stable group also scored relatively worse on executive function (Stroop inference test) and verbal memory (RAVLT). These findings are consistent with a previous study based on the 30-year follow-up of the overall CARDIA sample showing higher cumulative exposure to SBP or DBP had lower performance in memory; increasing cumulative exposure to SBP was also associated with executive and global domains in the 30-year follow-up.^5^

With the advancements in measuring and understanding mechanisms of BP-related pathology, additional longitudinal studies with repeated MRI imaging will aid in developing more precise treatment strategies for younger people, where the prevalence of hypertension is increasing. We noted that at-risk groups (ie, moderate-increasing and elevated-increasing) had disproportionately higher Black participants. It is known Black individuals have higher rates of hypertension, and exposure to risk factors for hypertension, such as disparities in socioeconomic environments and access to health care, may play a role.^36^ Further mechanistic studies with larger samples are needed to investigate whether race, sex, or other sociobehavioral factors modulate the effect of BP trajectory on brain outcomes. Even though our results suggest that the adverse associations of increasing BP with brain outcomes were not significant after adjustment of antihypertensive medication use, future work incorporating a time-varying design is needed to confirm the modulating effect of antihypertensive medication.

### Strengths and Limitations

Our study had several strengths. We studied the associations of young adulthood BP trajectories with brain MRI outcomes in midlife in a large, well-characterized biracial cohort. Such studies are needed, given the robust data suggesting elevated BP in midlife increases the risk for several late-life adverse brain outcomes. Moreover, we were able to estimate more robust trajectories based on the large number of cohort members who have been followed up to 25 years. These estimates were applied to a relatively large number of participants in the MRI data at year 25 and year 30 examinations.

This study also had several limitations. The MRI sample was a healthier group and included fewer Black participants than those who did not participate in the MRI substudy, which may have caused selection bias toward lower-risk participants. Even though more BP measurements would probably lead to more precise trajectory assignments, we included participants with 3 out of 8 BP measurements in the trajectory modeling. Nevertheless, in our study, the mean number of BP measurements is 7 in all trajectory groups, which indicates that the missingness of BP measurements was unlikely to change our results. A previous CARDIA study with a similar research design also found consistent trajectory assignments using imputed BP measurements.^12^

In addition, we did not examine the modulatory effect of sex or race on the association between trajectory group and brain outcomes because of a power issue (ie, small sample size for moderate-increasing and elevated-increasing groups). We found some discrepancies between results across 3 BP traits. MAP trajectories captured some of the integrated effects of SBP and DBP on vascular health but small sample size in certain trajectory groups may have limited our power to detect associations with SBP and DBP trajectories, and therefore these findings likely underestimated true associations. Residual confounding was possible due to measurement error and imperfectly self-reported health behaviors, particularly for physical activity.

## Conclusions

This study showed that moderate-increasing and elevated-increasing BP trajectories during early adulthood are associated with differences in structural brain outcomes as early as midlife. Taken together with evidence from late-life studies, preventing BP increases during young adulthood to middle age may be a promising strategy for prevention of dementia.

## Notes

Supplement.
eMethods.
**eFigure.** Sample Flowchart **eTable 1.** CARDIA Participant Characteristics for Individuals With and Without MRI Scan Data at Year 25 and Year 30 **eTable 2.** Systolic and Diastolic Blood Pressure and Mean Arterial Pressure at Each Study Examination by Blood Pressure Trajectory Group in the MRI Sample **eTable 3.** Systolic and Diastolic Blood Pressure and Mean Arterial Pressure at Each Study Examination by Blood Pressure Trajectory Group in the MRI Sample **eTable 4.** Prevalence of Hypertension at Each Study Examination by Blood Pressure Trajectory Group in the MRI Sample **eTable 5.** Brain Characteristics of the MRI Subset by SBP/DBP Trajectory Group—CARDIA Cohort: Year 25 and Year 30 Exam **eTable 6.** The Association of Systolic Blood Pressure (SBP) Trajectory With Brain Outcomes (Reference SBP Trajectory: Low-Stable, Overall n = 853) **eTable 7.** The Association of Diastolic Blood Pressure (DBP) Trajectory With Brain Outcomes (Reference DBP Trajectory: Low-Stable, Overall n = 853) **eTable 8.** The Association of Systolic Blood Pressure (SBP) Trajectory With Cognitive Function Outcomes at Year 30 Examination (Reference SBP Trajectory: Low-Stable, Overall n = 2736) **eTable 9.** The Association of Diastolic Blood Pressure (DBP) Trajectory With Cognitive Function Outcomes at Year 30 Examination (Reference DBP Trajectory: Low-Stable, Overall n = 2736) **eTable 10.** The Association of Mean Arterial Pressure (MAP) Trajectory With Cognitive Function Outcomes at Year 30 Examination (Reference MAP Trajectory: Low-Stable, Overall n = 2736) **eTable 11.** The Association of Systolic Blood Pressure (SBP) Trajectory With Brain Outcomes After Adjustment of Year 25 and Year 30 BP Measure (Reference SBP Trajectory: Low-Stable, Overall n = 853) **eTable 12.** The Association of Diastolic Blood Pressure (DBP) Trajectory With Brain Outcomes After Adjustment of Year 25 and Year 30 BP Measure (Reference DBP Trajectory: Low-Stable, Overall n = 853) **eTable 13.** The Association of Mean Arterial Pressure (MAP) Trajectory With Brain Outcomes After Adjustment of Year 25 and Year 30 BP Measure (Reference MAP Trajectory: Low-Stable, Overall n = 853)
eReferences.

## References

[1] Williamson JD, Pajewski NM, Auchus AP, ; SPRINT MIND Investigators for the SPRINT Research Group. Effect of intensive vs standard blood pressure control on probable dementia: a randomized clinical trial. JAMA. 2019;321(6):553-561. doi:10.1001/jama.2018.21442 30688979 PMC6439590

[2] Qiu C, Winblad B, Fratiglioni L. The age-dependent relation of blood pressure to cognitive function and dementia. Lancet Neurol. 2005;4(8):487-499. doi:10.1016/S1474-4422(05)70141-1 16033691

[3] Gottesman RF, Schneider AL, Albert M, . Midlife hypertension and 20-year cognitive change: the atherosclerosis risk in communities neurocognitive study. JAMA Neurol. 2014;71(10):1218-1227. doi:10.1001/jamaneurol.2014.1646 25090106 PMC4226067

[4] Power MC, Weuve J, Gagne JJ, McQueen MB, Viswanathan A, Blacker D. The association between blood pressure and incident Alzheimer disease: a systematic review and meta-analysis. Epidemiology. 2011;22(5):646-659. doi:10.1097/EDE.0b013e31822708b5 21705906 PMC3640480

[5] Mahinrad S, Kurian S, Garner CR, . Cumulative blood pressure exposure during young adulthood and mobility and cognitive function in midlife. Circulation. 2020;141(9):712-724. doi:10.1161/CIRCULATIONAHA.119.042502 31747780 PMC7135652

[6] Yaffe K, Vittinghoff E, Pletcher MJ, . Early adult to midlife cardiovascular risk factors and cognitive function. Circulation. 2014;129(15):1560-1567. doi:10.1161/CIRCULATIONAHA.113.004798 24687777 PMC4700881

[7] Walker KA, Sharrett AR, Wu A, . Association of midlife to late-life blood pressure patterns with incident dementia. JAMA. 2019;322(6):535-545. doi:10.1001/jama.2019.10575 31408138 PMC6692677

[8] McGrath ER, Beiser AS, DeCarli C, . Blood pressure from mid- to late life and risk of incident dementia. Neurology. 2017;89(24):2447-2454. doi:10.1212/WNL.0000000000004741 29117954 PMC5729797

[9] Power MC, Schneider AL, Wruck L, . Life-course blood pressure in relation to brain volumes. Alzheimers Dement. 2016;12(8):890-899. doi:10.1016/j.jalz.2016.03.012 27139841 PMC4980244

[10] Muller M, Sigurdsson S, Kjartansson O, ; Age, Gene/Environment Susceptibility-Reykjavik Study Investigators. Joint effect of mid- and late-life blood pressure on the brain: the AGES-Reykjavik study. Neurology. 2014;82(24):2187-2195. doi:10.1212/WNL.0000000000000517 24898928 PMC4113458

[11] Launer LJ, Lewis CE, Schreiner PJ, . Vascular factors and multiple measures of early brain health: CARDIA brain MRI study. PLoS One. 2015;10(3):e0122138. doi:10.1371/journal.pone.0122138 25812012 PMC4374951

[12] Allen NB, Siddique J, Wilkins JT, . Blood pressure trajectories in early adulthood and subclinical atherosclerosis in middle age. JAMA. 2014;311(5):490-497. doi:10.1001/jama.2013.285122 24496536 PMC4122296

[13] Glodzik L, Rusinek H, Pirraglia E, . Blood pressure decrease correlates with tau pathology and memory decline in hypertensive elderly. Neurobiol Aging. 2014;35(1):64-71. doi:10.1016/j.neurobiolaging.2013.06.011 23969178 PMC3799812

[14] Cherbuin N, Mortby ME, Janke AL, Sachdev PS, Abhayaratna WP, Anstey KJ. Blood pressure, brain structure, and cognition: opposite associations in men and women. Am J Hypertens. 2015;28(2):225-231. doi:10.1093/ajh/hpu120 25159080

[15] Allan CL, Zsoldos E, Filippini N, . Lifetime hypertension as a predictor of brain structure in older adults: cohort study with a 28-year follow-up. Br J Psychiatry. 2015;206(4):308-315. doi:10.1192/bjp.bp.114.153536 25497301 PMC4381190

[16] Friedman GD, Cutter GR, Donahue RP, . CARDIA: study design, recruitment, and some characteristics of the examined subjects. J Clin Epidemiol. 1988;41(11):1105-1116. doi:10.1016/0895-4356(88)90080-7 3204420

[17] Gunderson EP, Lewis CE, Tsai A-L, . A 20-year prospective study of childbearing and incidence of diabetes in young women, controlling for glycemia before conception: the Coronary Artery Risk Development in Young Adults (CARDIA) Study. Diabetes. 2007;56(12):2990-2996. doi:10.2337/db07-1024 17898128 PMC2952440

[18] Shen D, Davatzikos C. HAMMER: hierarchical attribute matching mechanism for elastic registration. IEEE Trans Med Imaging. 2002;21(11):1421-1439. doi:10.1109/TMI.2002.803111 12575879

[19] Lao Z, Shen D, Liu D, . Computer-assisted segmentation of white matter lesions in 3D MR images using support vector machine. Acad Radiol. 2008;15(3):300-313. doi:10.1016/j.acra.2007.10.012 18280928 PMC2528894

[20] Zacharaki EI, Kanterakis S, Bryan RN, Davatzikos C. Measuring brain lesion progression with a supervised tissue classification system. Springer; 2008:620-627. doi:10.1007/978-3-540-85988-8_74 18979798

[21] Goldszal AF, Davatzikos C, Pham DL, Yan MX, Bryan RN, Resnick SM. An image-processing system for qualitative and quantitative volumetric analysis of brain images. J Comput Assist Tomogr. 1998;22(5):827-837. doi:10.1097/00004728-199809000-00030 9754125

[22] Elbejjani M, Auer R, Dolui S, . Cigarette smoking and cerebral blood flow in a cohort of middle-aged adults. J Cereb Blood Flow Metab. 2019;39(7):1247-1257. doi:10.1177/0271678X18754973 29355449 PMC6668508

[23] Jacobs DR Jr, Hahn LP, Haskell WL, Pirie P, Sidney S. Validity and reliability of short physical activity history: CARDIA and the Minnesota Heart Health Program. J Cardiopulm Rehabil. 1989;9(11):448-459. doi:10.1097/00008483-198911000-00003 29657358 PMC5894828

[24] Jones BL, Nagin DS. Advances in group-based trajectory modeling and an SAS procedure for estimating them. Sociological Methods Research. 2007;35(4):542-571. doi:10.1177/0049124106292364

[25] Jones BL, Nagin DS, Roeder K. A SAS procedure based on mixture models for estimating developmental trajectories. Sociological Methods & Research. 2001;29(3):374-393. doi:10.1177/0049124101029003005

[26] Nagin DS, Tremblay RE. Analyzing developmental trajectories of distinct but related behaviors: a group-based method. Psychol Methods. 2001;6(1):18-34. doi:10.1037/1082-989X.6.1.18 11285809

[27] Nagin DS, Odgers CL. Group-based trajectory modeling in clinical research. Annu Rev Clin Psychol. 2010;6:109-138. doi:10.1146/annurev.clinpsy.121208.131413 20192788

[28] Nagin DS. Group-based modeling of development. Harvard University Press; 2005.

[29] Benjamini Y, Hochberg Y. Controlling the false discovery rate: a practical and powerful approach to multiple testing. Journal of the Royal Statistical Society: Series B (Methodological). 1995;57(1):289-300.

[30] Debette S, Seshadri S, Beiser A, . Midlife vascular risk factor exposure accelerates structural brain aging and cognitive decline. Neurology. 2011;77(5):461-468. doi:10.1212/WNL.0b013e318227b227 21810696 PMC3146307

[31] Gottesman RF, Coresh J, Catellier DJ, . Blood pressure and white-matter disease progression in a biethnic cohort: Atherosclerosis Risk in Communities (ARIC) study. Stroke. 2010;41(1):3-8. doi:10.1161/STROKEAHA.109.566992 19926835 PMC2803313

[32] Longstreth WT Jr, Arnold AM, Beauchamp NJ Jr, . Incidence, manifestations, and predictors of worsening white matter on serial cranial magnetic resonance imaging in the elderly: the Cardiovascular Health Study. Stroke. 2005;36(1):56-61. doi:10.1161/01.STR.0000149625.99732.69 15569873

[33] Marcus J, Gardener H, Rundek T, . Baseline and longitudinal increases in diastolic blood pressure are associated with greater white matter hyperintensity volume: the northern Manhattan study. Stroke. 2011;42(9):2639-2641. doi:10.1161/STROKEAHA.111.617571 21836088 PMC3189513

[34] Iadecola C, Davisson RL. Hypertension and cerebrovascular dysfunction. Cell Metab. 2008;7(6):476-484. doi:10.1016/j.cmet.2008.03.010 18522829 PMC2475602

[35] Schmidt R, Seiler S, Loitfelder M. Longitudinal change of small-vessel disease-related brain abnormalities. J Cereb Blood Flow Metab. 2016;36(1):26-39. doi:10.1038/jcbfm.2015.72 25899293 PMC4758559

[36] Carnethon MR, Pu J, Howard G, ; American Heart Association Council on Epidemiology and Prevention; Council on Cardiovascular Disease in the Young; Council on Cardiovascular and Stroke Nursing; Council on Clinical Cardiology; Council on Functional Genomics and Translational Biology; and Stroke Council. Cardiovascular health in African Americans: a scientific statement from the American Heart Association. Circulation. 2017;136(21):e393-e423. doi:10.1161/CIR.0000000000000534 29061565

