# Supplementary material for: Association of Early Adulthood 25-Year Blood Pressure Trajectories With Cerebral Lesions and Brain Structure in Midlife
Source: JAMA Netw Open. 2022 Mar 10;5(3):e221175. doi: 10.1001/jamanetworkopen.2022.1175 (PMC8914577; doi:10.1001/jamanetworkopen.2022.1175)
Supplement: Supplement. — eMethods. eFigure. Sample Flowchart eTable 1. CARDIA Participant Characteristics for Individuals With and Without MRI Scan Data at Year 25 and Year 30 eTable 2. Systolic and Diastolic Blood Pressure and Mean Arterial Pressure at Each Study Examination by Blood Pressure Trajectory Group in the MRI Sample eTable 3. Systolic and Diastolic Blood Pressure and Mean Arterial Pressure at Each Study Examination by Blood Pressure Trajectory Group in the MRI Sample eTable 4. Prevalence of Hypertension at Each Study Examination by Blood Pressure Trajectory Group in the MRI Sample eTable 5. Brain Characteristics of the MRI Subset by SBP/DBP Trajectory Group—CARDIA Cohort: Year 25 and Year 30 Exam eTable 6. The Association of Systolic Blood Pressure (SBP) Trajectory With Brain Outcomes (Reference SBP Trajectory: Low-Stable, Overall n = 853) eTable 7. The Association of Diastolic Blood Pressure (DBP) Trajectory With Brain Outcomes (Reference DBP Trajectory: Low-Stable, Overall n = 853) eTable 8. The Association of Systolic Blood Pressure (SBP) Trajectory With Cognitive Function Outcomes at Year 30 Examination (Reference SBP Trajectory: Low-Stable, Overall n = 2736) eTable 9. The Association of Diastolic Blood Pressure (DBP) Trajectory With Cognitive Function Outcomes at Year 30 Examination (Reference DBP Trajectory: Low-Stable, Overall n = 2736) eTable 10. The Association of Mean Arterial Pressure (MAP) Trajectory With Cognitive Function Outcomes at Year 30 Examination (Reference MAP Trajectory: Low-Stable, Overall n = 2736) eTable 11. The Association of Systolic Blood Pressure (SBP) Trajectory With Brain Outcomes After Adjustment of Year 25 and Year 30 BP Measure (Reference SBP Trajectory: Low-Stable, Overall n = 853) eTable 12. The Association of Diastolic Blood Pressure (DBP) Trajectory With Brain Outcomes After Adjustment of Year 25 and Year 30 BP Measure (Reference DBP Trajectory: Low-Stable, Overall n = 853) eTable 13. The Association of Mean Arterial Pressure (MAP) Trajecto [file jamanetwopen-e221175-s001.pdf]

Supplemental Online Content

Hu YH, Halstead MR, Bryan RN, et al. Association of early adulthood 25-year blood pressure trajectories to cerebral lesions and brain structure in midlife. *JAMA Netw Open.* 2022;5(3):e221175. doi:10.1001/jamanetworkopen.2022.1175

eMethods.

eFigure. Sample Flowchart

eTable 1. CARDIA Participant Characteristics for Individuals With and Without MRI Scan Data at Year 25 and Year 30

eTable 2. Systolic and Diastolic Blood Pressure and Mean Arterial Pressure at Each Study Examination by Blood Pressure Trajectory Group in the MRI Sample

eTable 3. Systolic and Diastolic Blood Pressure and Mean Arterial Pressure at Each Study Examination by Blood Pressure Trajectory Group in the MRI Sample

eTable 4. Prevalence of Hypertension at Each Study Examination by Blood Pressure Trajectory Group in the MRI Sample

eTable 5. Brain Characteristics of the MRI Subset by SBP/DBP Trajectory Group—CARDIA Cohort: Year 25 and Year 30 Exam

eTable 6. The Association of Systolic Blood Pressure (SBP) Trajectory With Brain Outcomes (Reference SBP Trajectory: Low-Stable, Overall n = 853)

eTable 7. The Association of Diastolic Blood Pressure (DBP) Trajectory With Brain Outcomes (Reference DBP Trajectory: Low-Stable, Overall n = 853)

eTable 8. The Association of Systolic Blood Pressure (SBP) Trajectory With Cognitive Function Outcomes at Year 30 Examination (Reference SBP Trajectory: Low-Stable, Overall n = 2736)

eTable 9. The Association of Diastolic Blood Pressure (DBP) Trajectory With Cognitive Function Outcomes at Year 30 Examination (Reference DBP Trajectory: Low-Stable, Overall n = 2736)

eTable 10. The Association of Mean Arterial Pressure (MAP) Trajectory With Cognitive Function Outcomes at Year 30 Examination (Reference MAP Trajectory: Low-Stable, Overall n = 2736)

eTable 11. The Association of Systolic Blood Pressure (SBP) Trajectory With Brain Outcomes After Adjustment of Year 25 and Year 30 BP Measure (Reference SBP Trajectory: Low-Stable, Overall n = 853)

© 2022 Hu YH et al. *JAMA Network Open.*

eTable 12. The Association of Diastolic Blood Pressure (DBP) Trajectory With Brain Outcomes After Adjustment of Year 25 and Year 30 BP Measure (Reference DBP Trajectory: Low-Stable, Overall n = 853)

eTable 13. The Association of Mean Arterial Pressure (MAP) Trajectory With Brain Outcomes After Adjustment of Year 25 and Year 30 BP Measure (Reference MAP Trajectory: Low-Stable, Overall n = 853)

eReferences.

This supplemental material has been provided by the authors to give readers additional information about their work.

© 2022 Hu YH et al. *JAMA Network Open.*

eMethods.

CARDIA Brain MRI sub-study

The sample for the CARDIA Brain magnetic resonance imaging (MRI) sub-study was initially enrolled at Y25 exam from 3 of the CARDIA field centers: Birmingham, Minneapolis, and Oakland. Each of the 3 centers had a target sample size for the sub-study, with the aim of achieving a balance within 4 strata of ethnicity/race (black/white) and sex (men/women). When the target sample size was reached, enrollment was ended. Exclusion criteria included a contraindication to MRI or a body size that was too large to enable the MRI examination. At year 25, 719 individuals from the CARDIA cohort underwent brain MRI examination. This same baseline sample was re-invited for a second scan in the Y30 follow-up. As some participants did not have a second exam (n=231), we recruited new participants (n=175) giving a total of 663; 488 had repeat MRIs. We merged the sample with good quality MRI scans to increase the sample size (n=885).

Details on the MRI hardware and quality control

All brain MRI scans were performed per CARDIA protocol using 3-Tesla magnetic resonance scanners, and standardized across machines using a common machine head phantom (Oakland: Siemens [Munich, Germany] 3T Tim Trio/VB 15 platform; Minneapolis: Siemens 3T Tim Trio/VB 15 platform; and Birmingham: Philips [Best, the Netherlands] 3T Achieva/2.6.3.6 platform).[1] Structural [3D sagittal T1, T2, and fluid-attenuated inversion recovery] and pseudo-continuous arterial spin labeling sequences were acquired. Brain MRI imaging analyses, quality control checks, and atlas registration were performed at the CARDIA Brain MRI Reading Center (Department of Radiology, University of Pennsylvania, Philadelphia, PA) according to standardized protocols as described previously.[1] Brain microstructural tissue integrity was estimated from axial diffusion tensor images using previously described parameters.[1]

Details on the combination of Y25 and Y30 MRI data

The brain magnetic resonance imaging (MRI) was conducted for a subset of CARDIA participants at exams of Y25 (n=719) and Y30 (n=663). Of the unique 885 subjects with MRI scans, 485 had repeated exams of good quality. To increase our sample size, we pooled the data from the 2 exams by calculating the weighted average for each brain measure. When combining the MRI measures (means), it seems appropriate to weight them according to the amount of noise (ex., due to machine error) associated with each. In addition, the MRI measures measured at Y30 exam may have larger variance than Y25 (biological portion). To get a weighted average, per sequence, we first estimated the noise and biological portions for each MRI outcomes based on the repeated data. The MRI measures at exams of Y25 and Y30 can be written as:

$$X_{i1} = U_i + T_{i1}$$
$$X_{i2} = AU_i + T_{i2}$$
$$U_i \sim N(0, \sigma^2) ; T_{i1} \sim N(0, \sigma^2/w) ; T_{i2} \sim N(0, \sigma^2/w)$$

We define X_{ij} as the observed trait value of individual i at the jth exam (i.e., X_{i1} is the MRI outcome at the exam Y25), U_i as the real trait value of individual i, and T_{ij} as the noise component of individual i at the jth exam. A is the weight that represents the unequal variances at the different time points (biological portion). All T_{ij} are independent from U_i and independent from one another. In addition, we assume U_i is normal with mean 0 and variance σ^2, and T_{ij} is normal with mean 0 and variance σ^2/w. And the weighted average was calculated as:

© 2022 Hu YH et al. *JAMA Network Open.*

$$\hat{U} = (wX_{i1} + wAX_{i2})/(1 + w + A^2w)$$

To get *w* and *A*, we first estimated the slope of the regression model $X_{i2} \sim X_{i1}$:

$$E\,[X_{i2}X_{i1}]/var(X_{i1}) = (A\sigma^2)/(\sigma^2 + \sigma^2/w) = Aw/(1 + w)$$

And then, we calculated the ratio of variances (δ):

$$var(X_{i2})/var(X_{i1}) = (A^2\sigma^2 + \sigma^2/w)/(\sigma^2 + \sigma^2/w) = (A^2w + 1)/(1 + w)$$

In short, we solved the quadratic equation to get *w* and *A*:

$$Aw/(1 + w) = Slope\ (\beta)$$

$$(A^2w + 1)/(1 + w) = variance\ ratio\ (\delta)$$

And *w* and *A* are equal to:

$$w = \frac{\delta - 1 - 2\beta^2 - \sqrt{(\delta - 1)^2 + (4\beta)^2}}{2(\beta^2 - \delta)}$$

$$A = \beta\frac{1 + w}{w}$$

The weighted average was calculated as the following formulas:

- Subjects with repeated MRI measures:

$$Weighted\ average = (wX_{i1} + wAX_{i2})/(1 + w + A^2w)$$

- Subjects with one-time point data at Y25:

$$Weighted\ average = (wX_{i1})/(1 + w)$$

- Subjects with one-time point data at Y30:

$$Weighted\ average = (wAX_{i2})/(1 + A^2w)$$

Correcting volume measures for intracranial volume differences between Y25 and Y30

There was a within person change in total intracranial volume (ICV), which should be constant over time. To account for this, the tissue volume needs to be benchmarked against the same standard. Therefore, all volume measures at the exam of Y30 were corrected by the following equation before calculating the weighted average.

$$Adjusted\ Y30\ volume\ measure = Raw\ Y30\ volume\ measure \times (Y25\ ICV/Y30\ ICV)$$

Details of group-based trajectory modeling

Group-based trajectory modeling (GBTM, SAS Proc Traj, v9.4) was used to identify unique polynomial functions that define both intercepts and slopes for each group trajectory. We identified 5 distinct trajectories for each blood pressure trait, including systolic blood pressure (SBP), diastolic blood pressure (DBP) and mean arterial pressure (MAP), across 25 years. The CARDIA has measured blood pressure at each of exam, with up to 8 BP measures. Blood pressure measured during pregnancy was removed from GBTM analysis. Subjects with at least 3 repeated BP measures from Y0 to Y25 were included in the trajectory modeling.

As reported in Allen et al.'s JAMA study,[2] we hypothesized 5 trajectory groups for each blood pressure trait. We then investigated models with a varying number of trajectory groups in a stepwise manner, starting with the highest polynomial (ex., cubic function) for each group trajectory. If the highest polynomial is not statistically significantly different from 0 ($p > .05$), we replaced it with a lower polynomial function (ex., quadratic or linear function).[3] The final number of trajectories were determined by the Bayesian Inclusion Criteria (BIC), with larger negative values for the BIC signifying better fit.

© 2022 Hu YH et al. *JAMA Network Open.*

To ensure that Proc Traj program accurately assigned each subject to the appropriate trajectory group, four diagnostic criteria were used to evaluate the adequacy of the selected models:[3] 1) the average posterior probability (AvePP) calculated for each trajectory group exceeded 0.7 threshold; 2) the estimated probability of group membership (π) should correspond closely to the proportion of the sample assigned to the group (number of people in that group / total number of subjects); 3) the confidence intervals around estimated group memberships (π) should be reasonably narrow; 4) the odds of correct classification (OCC) for each trajectory group exceeded the minimum threshold of 5, where OCC is defined by $[(AvePP/(1-AvePP)] / [\pi/(1-\pi)]$.

© 2022 Hu YH et al. *JAMA Network Open.*

eFigure. Sample Flowchart

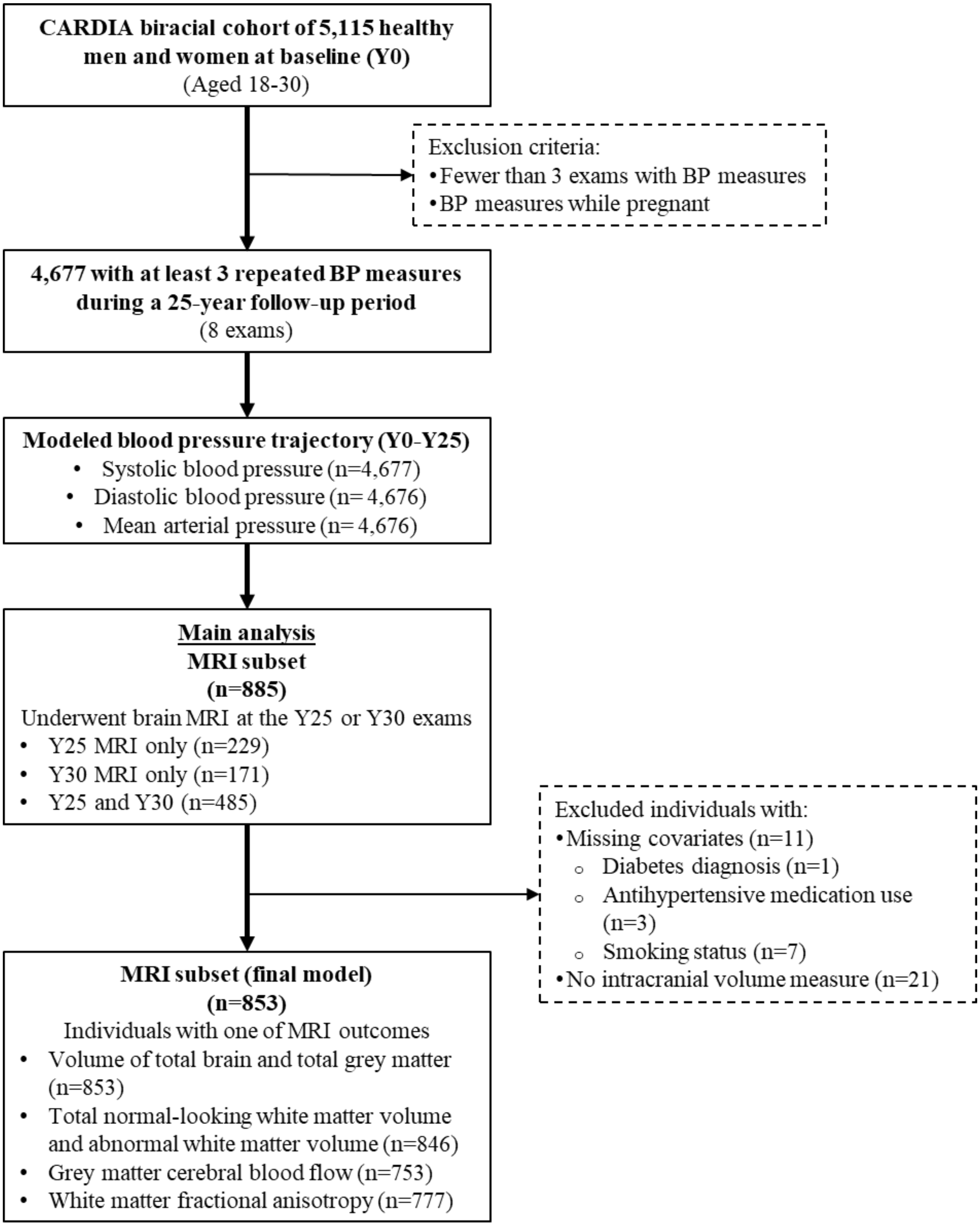

Figure S1. Sample flowchart

© 2022 Hu YH et al. *JAMA Network Open.*

eTable 1. CARDIA participant characteristics for individuals with and without MRI scan data at Year 25/Year 30 [a]

	Overall sample		Overall (n=4,677)				
			No MRI subset		MRI subset		*p*-value[d]
	(n=4,677)		(n=3,792)		(n=885)		
	No.(%)		No.(%)		No.(%)		
**0. Mean number of exams** (Range: 3-8)[b]	6.87(1.51)		6.68(1.58)		7.67(0.78)		<.0001
**1. Blood pressure trajectory**							
**SBP Trajectory Group**							0.02
Low-stable	1,071 (22.9)		858 (22.6)		213 (24.1)		
Moderate-gradual	2,122 (45.4)		1,713 (45.2)		409 (46.2)		
Moderate-increasing	333 (7.1)		280 (7.4)		53 (6.0)		
Elevated-stable	991 (21.2)		797 (21.0)		194 (21.9)		
Elevated-increasing	160 (3.4)		144 (3.8)		16 (1.8)		
**DBP Trajectory Group**							0.15
Low-stable	941 (20.1)		756 (19.9)		185 (20.9)		
Moderate-gradual	2,062 (44.1)		1,663 (43.9)		399 (45.1)		
Moderate-increasing	519 (11.1)		440 (11.6)		79 (8.9)		
Elevated-stable	940 (20.1)		753 (19.9)		187 (21.1)		
Elevated-increasing	214 (4.6)		179 (4.7)		35 (4.0)		
**MAP Trajectory Group**							0.18
Low-stable	917 (19.6)		730 (19.3)		187 (21.1)		
Moderate-gradual	2,056 (44.0)		1,671 (44.0)		385 (43.5)		
Moderate-increasing	439 (9.4)		368 (9.7)		71 (8.0)		
Elevated-stable	1,018 (21.8)		814 (21.5)		204 (23.1)		
Elevated-increasing	246 (5.2)		208 (5.5)		38 (4.3)		
**2. Sociodemographics**							
**Age**[b]	50.19(3.65)		50.15(3.67)		50.31(3.60)		0.24
**Male**	2,108 (45.1)		1,689 (44.5)		419 (47.3)		0.14
**Black**	2,343 (50.1)		1,977 (52.1)		366 (41.4)		<.0001
**Education**							<.0001
High school or less	1,489 (39.8)		1,140 (40.0)		349 (39.4)		
College	1,506 (40.3)		1,106 (38.8)		400 (45.2)		
Graduate school	743 (19.9)		607 (21.2)		136 (15.4)		
**3. Clinical measures**							
**Hypertension**[c]	1,997 (53.4)		1,560 (54.7)		437 (49.4)		0.006
**Taking antihypertensive Rx**	942 (27.1)		731 (28.2)		211 (23.9)		0.02
**BMI**							<.0001

© 2022 Hu YH et al. *JAMA Network Open.*

<25	880 (25.2)		627 (24.1)		253 (28.6)		
30 > BMI ≥ 25	1,094 (31.4)		784 (30.1)		310 (35.0)		
≥ 30	1,515 (43.4)		1,193 (45.8)		322 (36.4)		
**Diabetes**	568 (15.3)		452 (16.0)		116 (13.1)		0.04
**4. Behavior**							
**Current smoker**	584 (17.0)		436 (17.0)		148 (16.9)		0.97
**Alcohol use**	2,711 (78.2)		2,019 (78.3)		692 (78.2)		1.00
**Physical activity, median (IQR)**	276.0 (131.25-488.0)		270.0 (124.0-480.0)		291.0 (144.0-507.0)		0.07

Data source: The Coronary Artery Risk Development in Young Adults (CARDIA), 1985-2016
SBP=Systolic blood pressure; DBP=Diastolic blood pressure; MAP=Mean arterial pressure [(SBP*1/3)+(DBP*2/3)]; IQR=Interquartile range.
a: All covariates (i.e., sociodemographics and health condition) were collected at the Y25 exam. For participants with Y30 MRI only, missing covariates were updated using Y30 information.
b: Data was reported as mean and SD
c: SBP≥130 or DBP≥80 or taking antihypertensive medication
d: Differences in categorical variables assessed with chi-square and continuous variables with a non-parametric test for comparing means (or medians for physical activity).

© 2022 Hu YH et al. *JAMA Network Open.*

Table 2. Systolic and diastolic blood pressure and mean arterial pressure at each study exam by blood pressure trajectory group in the MRI sample								
	**Year 0**	**Year 2**	**Year 5**	**Year 7**	**Year 10**	**Year 15**	**Year 20**	**Year 25**
	(n=873)	(n=833)	(n=824)	(n=815)	(n=816)	(n=811)	(n=822)	(n=856)
	M(SD)	M(SD)	M(SD)	M(SD)	M(SD)	M(SD)	M(SD)	M(SD)
**SBP Trajectory Group**	**SBP (mm Hg)**							
Overall	110.35(10.51)	107.81(10.23)	107.70(11.04)	108.61(11.03)	109.36(11.75)	112.31(13.35)	114.27(13.34)	117.70(14.73)
Low-stable	101.05(7.33)	98.32(6.61)	97.19(6.40)	98.04(6.48)	97.65(6.93)	99.68(7.26)	101.13(6.47)	104.32(8.32)
Moderate-gradual	109.30(7.81)	106.88(7.08)	106.40(6.75)	106.85(6.54)	108.27(7.20)	111.32(8.81)	113.69(8.18)	117.71(9.95)
Moderate-increasing	112.65(7.59)	111.35(7.23)	111.98(10.90)	113.11(8.66)	115.74(9.84)	127.88(11.96)	135.26(16.71)	146.72(15.89)
Elevated-stable	120.86(7.49)	117.75(8.41)	118.42(8.48)	120.41(8.23)	120.82(8.95)	122.92(11.05)	123.31(10.19)	122.62(11.02)
Elevated-increasing	126.44(13.64)	124.73(13.47)	133.87(9.62)	133.43(12.21)	134.00(14.80)	140.79(14.38)	139.17(16.25)	143.96(15.43)
**DBP Trajectory Group**	**DBP (mm Hg)**							
Overall	68.98(9.20)	67.98(9.00)	69.69(9.75)	69.47(9.65)	72.18(9.62)	73.73(11.11)	70.89(10.77)	73.13(10.84)
Low-stable	62.24(7.32)	60.46(5.93)	60.20(6.71)	60.08(6.89)	62.38(6.37)	62.33(6.69)	59.58(5.96)	62.07(7.12)
Moderate-gradual	69.01(7.38)	68.12(8.40)	71.89(8.02)	71.89(8.05)	77.97(8.43)	84.04(9.53)	84.51(11.01)	87.81(8.43)
Moderate-increasing	67.29(7.24)	66.51(6.78)	68.29(6.63)	67.58(6.01)	70.33(6.12)	71.83(6.95)	69.95(6.94)	72.57(7.62)
Elevated-stable	76.53(7.31)	75.73(7.04)	77.73(7.32)	78.05(6.44)	80.37(6.97)	81.42(7.73)	76.20(7.89)	76.46(7.92)
Elevated-increasing	83.35(8.42)	81.15(10.12)	88.09(7.29)	86.88(10.56)	87.97(8.11)	95.00(9.72)	88.03(10.83)	88.84(11.52)
**MAP Trajectory Group**	**MAP (mm Hg)**							
Overall	82.77(8.60)	81.26(8.49)	82.36(9.47)	82.51(9.27)	84.57(9.77)	86.59(11.15)	85.34(11.17)	87.98(11.72)
Low-stable	75.56(6.30)	73.43(5.33)	72.96(5.80)	73.36(5.75)	74.50(5.64)	75.02(6.03)	73.61(5.46)	75.50(6.53)
Moderate-gradual	81.25(6.35)	79.83(5.91)	80.61(5.91)	80.30(5.34)	82.45(5.90)	84.70(6.66)	84.14(6.77)	87.48(7.79)
Moderate-increasing	83.22(7.37)	82.26(7.27)	84.35(8.07)	85.03(6.93)	90.21(8.83)	96.76(10.70)	100.12(12.31)	105.84(10.60)
Elevated-stable	89.50(6.46)	88.20(6.55)	90.11(6.72)	90.38(6.51)	92.51(7.01)	93.84(8.05)	91.05(7.97)	91.28(7.78)

© 2022 Hu YH et al. *JAMA Network Open.*

Elevated-increasing	96.81(8.90)	94.56(10.00)	101.07(7.76)	101.25(8.89)	101.24(9.77)	107.89(9.21)	103.24(12.77)	104.83(12.94)

Data source: The Coronary Artery Risk Development in Young Adults (CARDIA), 1985-2016
SBP=Systolic blood pressure; DBP=Diastolic blood pressure; MAP=Mean arterial pressure [(SBP*1/3)+(DBP*2/3)]; M=Mean; SD=Standard deviation.

© 2022 Hu YH et al. *JAMA Network Open.*

eTable 3. Systolic and diastolic blood pressure and mean arterial pressure at each study exam by blood pressure trajectory group in the MRI sample								
	**Year 0**	**Year 2**	**Year 5**	**Year 7**	**Year 10**	**Year 15**	**Year 20**	**Year 25**
	(n=873)	(n=833)	(n=824)	(n=815)	(n=816)	(n=811)	(n=822)	(n=856)
	M(SD)	M(SD)	M(SD)	M(SD)	M(SD)	M(SD)	M(SD)	M(SD)
**SBP Trajectory Group**	**SBP (mm Hg)**							
Overall	110.35(10.51)	107.81(10.23)	107.70(11.04)	108.61(11.03)	109.36(11.75)	112.31(13.35)	114.27(13.34)	117.70(14.73)
Low-stable	101.05(7.33)	98.32(6.61)	97.19(6.40)	98.04(6.48)	97.65(6.93)	99.68(7.26)	101.13(6.47)	104.32(8.32)
Moderate-gradual	109.30(7.81)	106.88(7.08)	106.40(6.75)	106.85(6.54)	108.27(7.20)	111.32(8.81)	113.69(8.18)	117.71(9.95)
Moderate-increasing	112.65(7.59)	111.35(7.23)	111.98(10.90)	113.11(8.66)	115.74(9.84)	127.88(11.96)	135.26(16.71)	146.72(15.89)
Elevated-stable	120.86(7.49)	117.75(8.41)	118.42(8.48)	120.41(8.23)	120.82(8.95)	122.92(11.05)	123.31(10.19)	122.62(11.02)
Elevated-increasing	126.44(13.64)	124.73(13.47)	133.87(9.62)	133.43(12.21)	134.00(14.80)	140.79(14.38)	139.17(16.25)	143.96(15.43)
**DBP Trajectory Group**	**DBP (mm Hg)**							
Overall	68.98(9.20)	67.98(9.00)	69.69(9.75)	69.47(9.65)	72.18(9.62)	73.73(11.11)	70.89(10.77)	73.13(10.84)
Low-stable	62.24(7.32)	60.46(5.93)	60.20(6.71)	60.08(6.89)	62.38(6.37)	62.33(6.69)	59.58(5.96)	62.07(7.12)
Moderate-gradual	69.01(7.38)	68.12(8.40)	71.89(8.02)	71.89(8.05)	77.97(8.43)	84.04(9.53)	84.51(11.01)	87.81(8.43)
Moderate-increasing	67.29(7.24)	66.51(6.78)	68.29(6.63)	67.58(6.01)	70.33(6.12)	71.83(6.95)	69.95(6.94)	72.57(7.62)
Elevated-stable	76.53(7.31)	75.73(7.04)	77.73(7.32)	78.05(6.44)	80.37(6.97)	81.42(7.73)	76.20(7.89)	76.46(7.92)
Elevated-increasing	83.35(8.42)	81.15(10.12)	88.09(7.29)	86.88(10.56)	87.97(8.11)	95.00(9.72)	88.03(10.83)	88.84(11.52)
**MAP Trajectory Group**	**MAP (mm Hg)**							
Overall	82.77(8.60)	81.26(8.49)	82.36(9.47)	82.51(9.27)	84.57(9.77)	86.59(11.15)	85.34(11.17)	87.98(11.72)
Low-stable	75.56(6.30)	73.43(5.33)	72.96(5.80)	73.36(5.75)	74.50(5.64)	75.02(6.03)	73.61(5.46)	75.50(6.53)
Moderate-gradual	81.25(6.35)	79.83(5.91)	80.61(5.91)	80.30(5.34)	82.45(5.90)	84.70(6.66)	84.14(6.77)	87.48(7.79)
Moderate-increasing	83.22(7.37)	82.26(7.27)	84.35(8.07)	85.03(6.93)	90.21(8.83)	96.76(10.70)	100.12(12.31)	105.84(10.60)
Elevated-stable	89.50(6.46)	88.20(6.55)	90.11(6.72)	90.38(6.51)	92.51(7.01)	93.84(8.05)	91.05(7.97)	91.28(7.78)

© 2022 Hu YH et al. *JAMA Network Open.*

Elevated-increasing	96.81(8.90)	94.56(10.00)	101.07(7.76)	101.25(8.89)	101.24(9.77)	107.89(9.21)	103.24(12.77)	104.83(12.94)
Data source: The Coronary Artery Risk Development in Young Adults (CARDIA), 1985-2016<br>SBP=Systolic blood pressure; DBP=Diastolic blood pressure; MAP=Mean arterial pressure [(SBP*1/3)+(DBP*2/3)]; M=Mean; SD=Standard deviation.								

© 2022 Hu YH et al. *JAMA Network Open.*

eTable 4. Prevalence of hypertension at each study exam by blood pressure trajectory group in the MRI sample[a]

	Year 0	Year 2	Year 5	Year 7	Year 10	Year 15	Year 20	Year 25
	(n=873)	(n=833)	(n=824)	(n=815)	(n=816)	(n=811)	(n=822)	(n=856)
	No.(%)	No.(%)	No.(%)	No.(%)	No.(%)	No.(%)	No.(%)	No.(%)
**Overall**	121 (13.9)	91 (10.9)	121 (14.7)	140 (17.2)	177 (21.7)	263 (32.4)	224 (27.2)	339 (39.6)
**SBP Trajectory Group**								
Low-stable	3 (1.4)	0 (0.0)	3 (1.5)	1 (0.5)	3 (1.5)	8 (3.9)	6 (3.0)	11 (5.3)
Moderate-gradual	30 (7.4)	24 (6.2)	25 (6.6)	26 (6.8)	50 (13.1)	87 (23.3)	72 (18.6)	143 (35.8)
Moderate-increasing	11 (21.6)	5 (10.4)	11 (22.4)	9 (20.0)	13 (31.0)	34 (79.1)	35 (76.1)	48 (98.0)
Elevated-stable	67 (35.1)	56 (30.6)	69 (37.5)	94 (51.9)	100 (55.2)	120 (67.8)	97 (55.7)	122 (65.9)
Elevated- increasing	10 (62.5)	6 (40.0)	13 (86.7)	10 (71.4)	11 (73.3)	14 (100.0)	14 (100.0)	15 (93.8)
**DBP Trajectory Group**								
Low-stable	0 (0.0)	0 (0.0)	0 (0.0)	0 (0.0)	0 (0.0)	2 (1.1)	2 (1.1)	14 (7.8)
Moderate-gradual	18 (4.6)	10 (2.7)	14 (3.7)	11 (3.0)	25 (6.7)	52 (14.2)	52 (13.9)	104 (26.7)
Moderate-increasing	9 (12.0)	4 (5.6)	11 (15.5)	14 (20.0)	29 (42.0)	57 (82.6)	57 (81.4)	71 (94.7)
Elevated-stable	69 (37.1)	55 (30.6)	68 (38.9)	88 (50.0)	95 (54.3)	121 (70.8)	87 (49.4)	119 (66.1)
Elevated- increasing	25 (73.5)	22 (66.7)	28 (87.5)	27 (81.8)	28 (87.5)	31 (100.0)	26 (96.3)	31 (96.9)
**MAP Trajectory Group**								
Low-stable	1 (0.5)	0 (0.0)	0 (0.0)	0 (0.0)	0 (0.0)	2 (1.1)	2 (1.1)	8 (4.4)
Moderate-gradual	19 (5.0)	9 (2.5)	13 (3.6)	9 (2.5)	24 (6.7)	55 (15.9)	49 (13.5)	105 (27.9)
Moderate-increasing	8 (11.8)	5 (7.6)	11 (16.4)	12 (19.4)	25 (39.1)	47 (75.8)	48 (78.7)	66 (98.5)
Elevated-stable	65 (32.2)	55 (28.1)	65 (34.6)	91 (47.9)	98 (51.3)	125 (66.1)	95 (50.0)	125 (63.8)
Elevated- increasing	28 (75.7)	22 (62.9)	32 (88.9)	28 (77.8)	30 (85.7)	34 (100.0)	30 (96.8)	35 (97.2)

Data source: The Coronary Artery Risk Development in Young Adults (CARDIA), 1985-2016
SBP=Systolic blood pressure; DBP=Diastolic blood pressure; MAP=Mean arterial pressure [(SBP*1/3)+(DBP*2/3)].
a: Hypertension defined as systolic blood pressure ≥130 or diastolic blood pressure≥80 or taking antihypertensive medication.

© 2022 Hu YH et al. *JAMA Network Open.*

eTable 5. Brain characteristics of the MRI subset by SBP/DBP trajectory group – CARDIA cohort: Year 25/Year 30 exam[a, b]							
	Overall	Trajectory group					
		Low-stable	Moderate-gradual	Moderate-increasing	Elevated-stable	Elevated-increasing	*p*-value[c]
	M(SD)	M(SD)	M(SD)	M(SD)	M(SD)	M(SD)	
<u>**SBP Trajectory Group**</u>							
**1. Brain volume measures (cm^3)**							
Valid n	853	207	398	50	183	15	
**Total brain**[d]	0.85(0.03)	0.85(0.03)	0.86(0.03)	0.85(0.03)	0.85(0.03)	0.84(0.04)	0.03
**Gray matter**[d]	0.46(0.02)	0.47(0.02)	0.47(0.02)	0.46(0.02)	0.46(0.02)	0.45(0.03)	0.002
Valid n	846	205	396	50	180	15	
**Normal-looking white matter**[d]	0.38(0.02)	0.38(0.02)	0.38(0.02)	0.38(0.02)	0.38(0.02)	0.38(0.02)	0.42
**Abnormal white matter**[e]	0.19(0.14)	0.18(0.12)	0.19(0.13)	0.27(0.25)	0.19(0.14)	0.31(0.27)	<.001
**2. White matter fractional anisotropy**	0.25(0.02)	0.26(0.02)	0.25(0.02)	0.24(0.02)	0.25(0.02)	0.24(0.02)	0.001
Valid n	777	187	367	42	168	13	
**3. Gray matter cerebral blood flow (mL/100g/min)**	29.72(7.22)	30.83(7.22)	30.20(7.43)	30.86(7.44)	27.34(6.35)	27.06(3.91)	<.001
Valid n	753	193	343	41	163	13	
<u>**DBP Trajectory Group**</u>							
**1. Brain volume measures (cm^3)**							
Valid n	853	181	385	73	181	33	
**Total brain**[d]	0.85(0.03)	0.85(0.02)	0.85(0.03)	0.86(0.03)	0.85(0.03)	0.85(0.03)	0.17
**Gray matter**[d]	0.46(0.02)	0.47(0.02)	0.47(0.02)	0.46(0.02)	0.46(0.02)	0.46(0.03)	0.05
Valid n	846	180	382	72	179	33	
**Normal-looking white matter**[d]	0.38(0.02)	0.38(0.01)	0.38(0.02)	0.38(0.01)	0.38(0.02)	0.38(0.02)	0.69
**Abnormal white matter**[e]	0.19(0.14)	0.19(0.12)	0.18(0.13)	0.23(0.19)	0.19(0.15)	0.26(0.19)	0.007
**2. White matter fractional anisotropy**	0.25(0.02)	0.26(0.02)	0.25(0.02)	0.25(0.02)	0.25(0.02)	0.25(0.02)	0.005

© 2022 Hu YH et al. *JAMA Network Open.*

Valid n	777		166		356		62		163		30	
**3. Gray matter cerebral blood flow (mL/100g/min)**	29.72(7.22)		30.64(7.62)		30.11(7.36)		29.81(6.35)		28.25(6.85)		27.08(5.24)	0.008
Valid n	753		169		344		64		147		29	

Data source: The Coronary Artery Risk Development in Young Adults (CARDIA), 1985-2016
SBP=systolic blood pressure; DBP=diastolic blood pressure.
a: Based on the cases with complete data on the outcome and all covariates.
b: Data is reported as mean (M) and standard deviation (SD).
c: p-value was based on one-way analysis of variance test.
d: Reported as brain volume to intracranial volume ratio
e: Log transformation was applied due to skew distribution.

© 2022 Hu YH et al. *JAMA Network Open.*

eTable 6. The association of systolic blood pressure (SBP) trajectory with brain outcomes (reference SBP trajectory: Low-stable, overall n=853) [a,b,c,d]									
	Low-stable (n=207)	Moderate-gradual (n=398)		Moderate-increasing (n=50)		Elevated-stable (n=183)		Elevated-increasing (n=15)	
	Mean[f]	β (95% CI)	*q*	β (95% CI)	*q*	β (95% CI)	*q*	β (95% CI)	*q*
**Model 1 estimations**									
Total brain volume	-0.06	0.03 (-0.02, 0.08)	0.34	-0.09 (-0.18, 0.01)	0.14	-0.02 (-0.09, 0.05)	0.68	-0.18 (-0.34, -0.02)	0.06
Total gray matter volume	-0.08	0.05 (-0.03, 0.13)	0.30	-0.12 (-0.25, 0.02)	0.18	-0.008 (-0.11, 0.09)	0.90	-0.31 (-0.54, -0.08)	0.03
Normal white matter volume	-0.04	0.009 (-0.05, 0.07)	0.81	-0.07 (-0.18, 0.04)	0.28	-0.02 (-0.10, 0.06)	0.67	-0.05 (-0.23, 0.14)	0.68
Abnormal white matter volume[e]	0.18	0.14 (-0.03, 0.31)	0.18	0.74 (0.43, 1.04)	<.001	0.24 (0.02, 0.47)	0.08	1.11 (0.59, 1.64)	0.001
White matter fractional anisotropy	0.23	-0.12 (-0.30, 0.05)	0.26	-0.38 (-0.70, -0.05)	0.06	-0.15 (-0.38, 0.08)	0.28	-0.58 (-1.12, -0.03)	0.08
Gray matter cerebral blood flow	0.45	0.007 (-0.16, 0.18)	0.93	-0.08 (-0.39, 0.23)	0.68	-0.18 (-0.40, 0.04)	0.18	-0.32 (-0.84, 0.20)	0.31
**Model 2 estimations**									
Total brain volume	0.01	0.03 (-0.02, 0.09)	0.34	-0.07 (-0.17, 0.03)	0.31	-0.003 (-0.07, 0.07)	0.92	-0.16 (-0.32, 0.00)	0.17
Total gray matter volume	-0.02	0.05 (-0.02, 0.13)	0.32	-0.08 (-0.22, 0.05)	0.34	0.01 (-0.09, 0.11)	0.86	-0.28 (-0.52, -0.05)	0.09
Normal white matter volume	0.02	0.01 (-0.05, 0.07)	0.79	-0.07 (-0.18, 0.04)	0.34	-0.02 (-0.10, 0.07)	0.79	-0.03 (-0.22, 0.15)	0.80
Abnormal white matter volume[e]	0.19	0.14 (-0.04, 0.31)	0.25	0.72 (0.40, 1.03)	<.001	0.22 (-0.01, 0.45)	0.18	1.05 (0.53, 1.58)	0.002
White matter fractional anisotropy	0.24	-0.11 (-0.28, 0.07)	0.34	-0.31 (-0.64, 0.02)	0.18	-0.09 (-0.33, 0.14)	0.55	-0.50 (-1.04, 0.04)	0.18
Gray matter cerebral blood flow	0.68	0.05 (-0.12, 0.22)	0.67	0.02 (-0.29, 0.34)	0.91	-0.11 (-0.33, 0.11)	0.45	-0.32 (-0.84, 0.19)	0.34
**Model 3 estimations**									
Total brain volume	0.01	0.04 (-0.01, 0.10)	0.44	-0.05 (-0.15, 0.05)	0.57	0.01 (-0.06, 0.09)	0.82	-0.13 (-0.30, 0.03)	0.44
Total gray matter volume	-0.01	0.06 (-0.02, 0.14)	0.44	-0.06 (-0.21, 0.08)	0.60	0.03 (-0.07, 0.14)	0.65	-0.25 (-0.49, -0.01)	0.31
Normal white matter volume	0.03	0.02 (-0.04, 0.08)	0.65	-0.05 (-0.16, 0.06)	0.60	0.001 (-0.09, 0.09)	0.99	-0.007 (-0.20, 0.18)	0.99

© 2022 Hu YH et al. *JAMA Network Open.*

Abnormal white matter volume[e]		0.18		0.12 (-0.06, 0.30)	0.44		0.67 (0.35, 0.99)	0.002		0.18 (-0.07, 0.42)	0.44		0.99 (0.45, 1.52)	0.006
White matter fractional anisotropy		0.27		-0.07 (-0.25, 0.10)	0.60		-0.23 (-0.56, 0.11)	0.44		-0.009 (-0.25, 0.24)	0.99		-0.38 (-0.93, 0.17)	0.44
Gray matter cerebral blood flow		0.68		0.06 (-0.11, 0.23)	0.65		0.03 (-0.29, 0.35)	0.95		-0.10 (-0.33, 0.14)	0.60		-0.30 (-0.83, 0.23)	0.52

Data source: The Coronary Artery Risk Development in Young Adults (CARDIA), 1985-2016
CI=confidence interval.
a: q-value was calculated using Benjamini-Hochberg method for the adjustment of multiple comparison. q ≤0.1 was considered statistically significant.
b: For the volume of total brain, gray matter and hippocampus, n= 853; normal-looking whiter matter and abnormal white matter volume, n=846; white matter fractional anisotropy and mean diffusivity, n=777; and cerebral blood flow n=753.
c: All outcomes were standardized to Z-score values.
d: All models were adjusted for age, sex, race, education, and field center. In Model 2, BMI, diabetes, physical activity level (log-transformed), current smoking status, and alcohol use were added. Model 3 was additionally adjusted for antihypertensive medication. Tissue volume measures models additionally adjusted for total intracranial volume; white matter fractional anisotropy and gray matter cerebral blood flow models additionally adjusted for total brain volume.
e: Abnormal white matter volume was log-transformed before standardizing to Z-score values.
f: Adjusted means based on the estimated model when intracranial volume/total brain volume was standardized, and age and physical activity level centered.

© 2022 Hu YH et al. *JAMA Network Open.*

eTable 7. The association of diastolic blood pressure (DBP) trajectory with brain outcomes (reference DBP trajectory: Low-stable, overall n=853) [a,b,c,d]									
	Low-stable (n=181)	Moderate-gradual (n=385)		Moderate-increasing (n=73)		Elevated-stable (n=181)		Elevated-increasing (n=33)	
	Mean[f]	β (95% CI)	*q*	β (95% CI)	*q*	β (95% CI)	*q*	β (95% CI)	*q*
**Model 1 estimations**									
Total brain volume	-0.04	-0.004 (-0.06, 0.05)	0.89	-0.04 (-0.13, 0.04)	0.46	-0.03 (-0.10, 0.04)	0.47	-0.11 (-0.23, 0.01)	0.15
Total gray matter volume	-0.07	0.02 (-0.06, 0.11)	0.64	-0.05 (-0.18, 0.07)	0.51	-0.01 (-0.11, 0.09)	0.89	-0.18 (-0.35, -0.01)	0.11
Normal white matter volume	-0.02	-0.03 (-0.09, 0.03)	0.47	-0.04 (-0.14, 0.06)	0.48	-0.06 (-0.13, 0.02)	0.24	-0.04 (-0.17, 0.10)	0.66
Abnormal white matter volume[e]	0.30	-0.09 (-0.27, 0.09)	0.47	0.21 (-0.08, 0.49)	0.24	-0.02 (-0.24, 0.20)	0.89	0.51 (0.13, 0.89)	0.06
White matter fractional anisotropy	0.26	-0.09 (-0.27, 0.09)	0.46	-0.31 (-0.60, -0.02)	0.11	-0.23 (-0.44, -0.01)	0.11	-0.3 (-0.69, 0.08)	0.23
Gray matter cerebral blood flow	0.53	-0.08 (-0.25, 0.09)	0.47	-0.23 (-0.50, 0.04)	0.21	-0.23 (-0.44, -0.02)	0.10	-0.48 (-0.85, -0.11)	0.06
**Model 2 estimations**									
Total brain volume	0.03	-0.006 (-0.06, 0.05)	0.86	-0.04 (-0.12, 0.05)	0.56	-0.03 (-0.10, 0.04)	0.54	-0.10 (-0.22, 0.01)	0.29
Total gray matter volume	-0.01	0.02 (-0.06, 0.10)	0.68	-0.04 (-0.17, 0.09)	0.66	-0.009 (-0.11, 0.09)	0.86	-0.17 (-0.34, -0.00)	0.23
Normal white matter volume	0.05	-0.03 (-0.10, 0.03)	0.52	-0.04 (-0.14, 0.06)	0.58	-0.05 (-0.13, 0.02)	0.42	-0.02 (-0.16, 0.11)	0.78
Abnormal white matter volume[e]	0.30	-0.09 (-0.27, 0.09)	0.52	0.19 (-0.11, 0.48)	0.42	-0.03 (-0.26, 0.19)	0.81	0.47 (0.09, 0.86)	0.15

© 2022 Hu YH et al. *JAMA Network Open.*

White matter fractional anisotropy		0.25		-0.09 (-0.27, 0.09)	0.53		-0.26 (-0.56, 0.03)	0.29		-0.20 (-0.42, 0.02)	0.29		-0.25 (-0.63, 0.14)	0.42
Gray matter cerebral blood flow		0.74		-0.03 (-0.21, 0.14)	0.78		-0.13 (-0.40, 0.15)	0.53		-0.17 (-0.38, 0.05)	0.38		-0.41 (-0.78, -0.04)	0.23
**Model 3 estimations**														
Total brain volume		0.03		-0.003 (-0.06, 0.05)	0.95		-0.02 (-0.11, 0.07)	0.82		-0.02 (-0.09, 0.05)	0.82		-0.08 (-0.20, 0.04)	0.61
Total gray matter volume		-0.001		0.03 (-0.06, 0.11)	0.75		-0.02 (-0.16, 0.11)	0.83		0.005 (-0.10, 0.11)	0.95		-0.15 (-0.33, 0.03)	0.58
Normal white matter volume		0.05		-0.03 (-0.09, 0.04)	0.68		-0.02 (-0.13, 0.08)	0.82		-0.04 (-0.12, 0.04)	0.68		-0.001 (-0.14, 0.14)	0.99
Abnormal white matter volume[e]		0.27		-0.10 (-0.29, 0.08)	0.68		0.13 (-0.17, 0.42)	0.68		-0.09 (-0.32, 0.14)	0.68		0.38 (-0.02, 0.78)	0.47
White matter fractional anisotropy		0.27		-0.08 (-0.26, 0.11)	0.68		-0.21 (-0.51, 0.10)	0.61		-0.14 (-0.37, 0.09)	0.61		-0.15 (-0.55, 0.25)	0.68
Gray matter cerebral blood flow		0.74		-0.03 (-0.21, 0.14)	0.83		-0.13 (-0.42, 0.15)	0.68		-0.17 (-0.39, 0.05)	0.61		-0.42 (-0.81, -0.03)	0.31

Data source: The Coronary Artery Risk Development in Young Adults (CARDIA), 1985-2016
CI=confidence interval.
a: q-value was calculated using Benjamini-Hochberg method for the adjustment of multiple comparison. q ≤0.1 was considered statistically significant.
b: For the volume of total brain, gray matter and hippocampus, n= 853; normal-looking whiter matter and abnormal white matter volume, n=846; white matter fractional anisotropy and mean diffusivity, n=777; and cerebral blood flow n=753.
c: All outcomes were standardized to Z-score values.
d: All models were adjusted for age, sex, race, education, and field center. In Model 2, BMI, diabetes, physical activity level (log-transformed), current smoking status, and alcohol use were added. Model 3 was additionally adjusted for antihypertensive medication. Tissue volume measures models additionally adjusted for total intracranial volume; white matter fractional anisotropy and gray matter cerebral blood flow models additionally adjusted for total brain volume.
e: Abnormal white matter volume was log-transformed before standardizing to Z-score values.
f: Adjusted means based on the estimated model when intracranial volume/total brain volume was standardized, and age and physical activity level centered.

© 2022 Hu YH et al. *JAMA Network Open.*

eTable 8. The association of systolic blood pressure (SBP) trajectory with cognitive function outcomes at Y30 exam (reference SBP trajectory: Low-stable, overall n=2,736) [a,b,c,d]

	Low-stable (n=705)	Moderate-gradual (n=1,225)		Moderate-increasing (n=197)		Elevated-stable (n=538)		Elevated-increasing (n=71)	
	Mean[d]	β (95% CI)	*q*	β (95% CI)	*q*	β (95% CI)	*q*	β (95% CI)	*q*
**Model 1 estimations**									
RAVLT	0.66	-0.11 (-0.20, -0.03)	0.04	-0.23 (-0.38, -0.09)	0.01	-0.18 (-0.29, -0.06)	0.01	-0.40 (-0.63, -0.18)	0.005
DSST	0.25	-0.04 (-0.13, 0.05)	0.45	-0.18 (-0.33, -0.04)	0.04	-0.19 (-0.31, -0.08)	0.007	-0.37 (-0.59, -0.15)	0.008
Stroop test	-0.27	0.07 (-0.02, 0.16)	0.22	0.24 (0.08, 0.39)	0.01	0.16 (0.04, 0.28)	0.03	0.28 (0.04, 0.52)	0.06
**Model 2 estimations**									
RAVLT	0.60	-0.11 (-0.19, -0.02)	0.09	-0.20 (-0.35, -0.05)	0.08	-0.14 (-0.25, -0.02)	0.09	-0.31 (-0.54, -0.09)	0.08
DSST	0.20	-0.02 (-0.11, 0.07)	0.76	-0.12 (-0.26, 0.03)	0.25	-0.12 (-0.23, -0.00)	0.17	-0.22 (-0.44, 0.00)	0.17
Stroop test	-0.22	0.06 (-0.04, 0.15)	0.34	0.19 (0.03, 0.34)	0.09	0.11 (-0.01, 0.23)	0.19	0.16 (-0.08, 0.40)	0.32
**Model 3 estimations**									
RAVLT	0.61	-0.09 (-0.18, 0.00)	0.33	-0.16 (-0.31, -0.00)	0.31	-0.09 (-0.21, 0.03)	0.44	-0.24 (-0.48, -0.01)	0.31
DSST	0.21	-0.002 (-0.09, 0.09)	0.99	-0.08 (-0.22, 0.07)	0.57	-0.07 (-0.19, 0.05)	0.48	-0.15 (-0.38, 0.08)	0.46
Stroop test	-0.23	0.03 (-0.06, 0.13)	0.65	0.14 (-0.02, 0.30)	0.44	0.06 (-0.07, 0.19)	0.60	0.08 (-0.16, 0.33)	0.65

Data source: The Coronary Artery Risk Development in Young Adults (CARDIA), 1985-2016
Abbreviations: RAVLT=Rey Auditory Verbal Learning Test; DSST=Digital symbol substitution test; CI=confidence interval.
a: q-value was calculated using Benjamini-Hochberg method for the adjustment of multiple comparison. q ≤0.1 was considered statistically significant.
b: All outcomes were standardized to Z-score values.
c: All models were adjusted for age, sex, race, education, and field center. In Model 2, BMI, diabetes, physical activity level (log-transformed), current smoking status, and alcohol use were added. Model 3 was additionally adjusted for antihypertensive medication.
d: Adjusted means based on the estimated model when intracranial volume/total brain volume was standardized, and age and physical activity level centered.

© 2022 Hu YH et al. *JAMA Network Open.*

eTable 9. The association of diastolic blood pressure (DBP) trajectory with cognitive function outcomes at Y30 exam (reference DBP trajectory: Low-stable, overall n=2736) [a,b,c,d]

	Low-stable (n=587)	Moderate-gradual (n=1,205)		Moderate-increasing (n=309)		Elevated-stable (n=531)		Elevated-increasing (n=104)	
	Mean[d]	β (95% CI)	*q*	β (95% CI)	*q*	β (95% CI)	*q*	β (95% CI)	*q*
**Model 1 estimations**									
RAVLT	0.66	-0.07 (-0.16, 0.02)	0.22	-0.24 (-0.37, -0.11)	0.004	-0.14 (-0.25, -0.03)	0.06	-0.37 (-0.56, -0.18)	0.003
DSST	0.24	0.02 (-0.07, 0.11)	0.69	-0.14 (-0.27, -0.01)	0.10	-0.13 (-0.24, -0.02)	0.08	-0.24 (-0.43, -0.05)	0.06
Stroop test	-0.28	0.07 (-0.02, 0.17)	0.24	0.18 (0.04, 0.32)	0.06	0.10 (-0.02, 0.22)	0.21	0.45 (0.24, 0.65)	0.001
**Model 2 estimations**									
RAVLT	0.60	-0.07 (-0.16, 0.02)	0.38	-0.20 (-0.33, -0.07)	0.04	-0.12 (-0.23, -0.00)	0.23	-0.33 (-0.52, -0.14)	0.02
DSST	0.18	0.05 (-0.04, 0.14)	0.52	-0.05 (-0.18, 0.08)	0.56	-0.07 (-0.18, 0.04)	0.42	-0.14 (-0.33, 0.05)	0.38
Stroop test	-0.23	0.06 (-0.04, 0.15)	0.46	0.12 (-0.02, 0.26)	0.29	0.06 (-0.06, 0.18)	0.52	0.39 (0.18, 0.59)	0.008
**Model 3 estimations**									
RAVLT	0.61	-0.06 (-0.15, 0.03)	0.61	-0.16 (-0.30, -0.02)	0.25	-0.08 (-0.20, 0.03)	0.61	-0.27 (-0.48, -0.07)	0.13
DSST	0.19	0.06 (-0.03, 0.14)	0.61	-0.01 (-0.14, 0.12)	0.95	-0.04 (-0.15, 0.08)	0.75	-0.08 (-0.28, 0.11)	0.68
Stroop test	-0.23	0.05 (-0.05, 0.14)	0.68	0.08 (-0.07, 0.22)	0.68	0.02 (-0.10, 0.15)	0.83	0.32 (0.11, 0.54)	0.11

Data source: The Coronary Artery Risk Development in Young Adults (CARDIA), 1985-2016
Abbreviations: RAVLT=Rey Auditory Verbal Learning Test; DSST=Digital symbol substitution test; CI=confidence interval.
a: q-value was calculated using Benjamini-Hochberg method for the adjustment of multiple comparison. q ≤0.1 was considered statistically significant.
b: All outcomes were standardized to Z-score values.

© 2022 Hu YH et al. *JAMA Network Open.*

c: All models were adjusted for age, sex, race, education, and field center. In Model 2, BMI, diabetes, physical activity level (log-transformed), current smoking status, and alcohol use were added. Model 3 was additionally adjusted for antihypertensive medication.
d: Adjusted means based on the estimated model when intracranial volume/total brain volume was standardized, and age and physical activity level centered.

© 2022 Hu YH et al. *JAMA Network Open.*

eTable 10. The association of mean arterial pressure (MAP) trajectory with cognitive function outcomes at Y30 exam (reference MAP trajectory: Low-stable, overall n=2736) [a,b,c,d]

	Low-stable (n=598)	Moderate-gradual (n=1,184)		Moderate-increasing (n=267)		Elevated-stable (n=567)		Elevated-increasing (n=120)	
	Mean[d]	β (95% CI)	*q*	β (95% CI)	*q*	β (95% CI)	*q*	β (95% CI)	*q*
**Model 1 estimations**									
RAVLT	0.68	-0.12 (-0.21, -0.03)	0.02	-0.31 (-0.44, -0.17)	<.001	-0.16 (-0.27, -0.05)	0.02	-0.33 (-0.52, -0.15)	0.004
DSST	0.26	-0.03 (-0.12, 0.06)	0.74	-0.21 (-0.35, -0.08)	0.01	-0.17 (-0.28, -0.06)	0.01	-0.29 (-0.47, -0.11)	0.01
Stroop test	-0.27	0.05 (-0.05, 0.15)	0.53	0.23 (0.08, 0.37)	0.01	0.17 (0.05, 0.29)	0.02	0.30 (0.10, 0.49)	0.01
**Model 2 estimations**									
RAVLT	0.61	-0.11 (-0.20, -0.02)	0.11	-0.26 (-0.40, -0.12)	0.007	-0.13 (-0.25, -0.02)	0.11	-0.28 (-0.46, -0.09)	0.03
DSST	0.20	0.01 (-0.08, 0.10)	0.97	-0.12 (-0.26, 0.01)	0.17	-0.1 (-0.22, 0.01)	0.17	-0.16 (-0.35, 0.02)	0.17
Stroop test	-0.21	0.02 (-0.07, 0.12)	0.89	0.16 (0.02, 0.31)	0.11	0.12 (0.00, 0.24)	0.14	0.21 (0.02, 0.41)	0.12
**Model 3 estimations**									
RAVLT	0.62	-0.10 (-0.19, -0.01)	0.23	-0.22 (-0.36, -0.08)	0.04	-0.10 (-0.22, 0.02)	0.43	-0.21 (-0.41, -0.02)	0.23
DSST	0.20	0.02 (-0.07, 0.11)	0.88	-0.08 (-0.22, 0.06)	0.55	-0.07 (-0.18, 0.05)	0.55	-0.10 (-0.29, 0.09)	0.58
Stroop test	-0.22	0.01 (-0.09, 0.11)	0.91	0.12 (-0.03, 0.27)	0.43	0.08 (-0.04, 0.21)	0.50	0.15 (-0.06, 0.36)	0.46

Data source: The Coronary Artery Risk Development in Young Adults (CARDIA), 1985-2016
Abbreviations: RAVLT=Rey Auditory Verbal Learning Test; DSST=Digital symbol substitution test; CI=confidence interval.
a: q-value was calculated using Benjamini-Hochberg method for the adjustment of multiple comparison. q ≤0.1 was considered statistically significant.
b: All outcomes were standardized to Z-score values.
c: All models were adjusted for age, sex, race, education, and field center. In Model 2, BMI, diabetes, physical activity level (log-transformed), current smoking status, and alcohol use were added. Model 3 was additionally adjusted for antihypertensive medication.
d: Adjusted means based on the estimated model when intracranial volume/total brain volume was standardized, and age and physical activity level centered.

© 2022 Hu YH et al. *JAMA Network Open.*

eTable 11. The association of systolic blood pressure (SBP) trajectory with brain outcomes after adjustment of Y25/Y30 BP measure (reference SBP trajectory: Low-stable, overall n=853) [a,b,c,d]

	Low-stable (n=207)	Moderate-gradual (n=398)		Moderate-increasing (n=50)		Elevated-stable (n=183)		Elevated-increasing (n=15)	
	Mean[f]	β (95% CI)	*q*	β (95% CI)	*q*	β (95% CI)	*q*	β (95% CI)	*q*
**Model 1 estimations**									
Total brain volume	-0.05	0.02 (-0.04, 0.08)	0.65	-0.12 (-0.24, 0.01)	0.22	-0.03 (-0.11, 0.05)	0.63	-0.21 (-0.39, -0.03)	0.11
Total gray matter volume	-0.10	0.06 (-0.02, 0.15)	0.33	-0.07 (-0.25, 0.11)	0.63	0.01 (-0.10, 0.12)	0.91	-0.27 (-0.53, -0.01)	0.17
Normal white matter volume	-0.003	-0.02 (-0.09, 0.04)	0.65	-0.18 (-0.32, -0.03)	0.09	-0.07 (-0.16, 0.02)	0.31	-0.15 (-0.35, 0.05)	0.33
Abnormal white matter volume[e]	0.24	0.09 (-0.10, 0.28)	0.56	0.58 (0.18, 0.99)	0.08	0.18 (-0.07, 0.43)	0.33	0.97 (0.39, 1.55)	0.04
White matter fractional anisotropy	0.15	-0.05 (-0.24, 0.14)	0.77	-0.14 (-0.57, 0.28)	0.65	-0.05 (-0.31, 0.21)	0.84	-0.35 (-0.95, 0.26)	0.50
Gray matter cerebral blood flow	0.31	0.14 (-0.04, 0.33)	0.31	0.35 (-0.06, 0.76)	0.27	0 (-0.24, 0.25)	0.99	0.1 (-0.48, 0.67)	0.84
**Model 2 estimations**									
Total brain volume	0.02	0.02 (-0.04, 0.08)	0.79	-0.11 (-0.23, 0.01)	0.29	-0.02 (-0.10, 0.06)	0.81	-0.20 (-0.38, -0.02)	0.25
Total gray matter volume	-0.03	0.06 (-0.02, 0.15)	0.38	-0.06 (-0.24, 0.12)	0.793	0.02 (-0.09, 0.14)	0.87	-0.26 (-0.52, 0.004)	0.28
Normal white matter volume	0.07	-0.02 (-0.09, 0.05)	0.79	-0.18 (-0.32, -0.03)	0.19	-0.06 (-0.15, 0.03)	0.38	-0.14 (-0.35, 0.06)	0.38
Abnormal white matter volume[e]	0.24	0.10 (-0.09, 0.29)	0.57	0.59 (0.19, 1.00)	0.08	0.17 (-0.09, 0.42)	0.40	0.93 (0.35, 1.51)	0.06
White matter fractional anisotropy	0.17	-0.05 (-0.24, 0.14)	0.81	-0.12 (-0.54, 0.30)	0.81	-0.01 (-0.27, 0.25)	0.95	-0.31 (-0.91, 0.30)	0.57
Gray matter cerebral blood flow	0.54	0.17 (-0.01, 0.36)	0.28	0.40 (-0.00, 0.81)	0.28	0.05 (-0.20, 0.29)	0.87	0.05 (-0.52, 0.62)	0.95
**Model 3 estimations**									
Total brain volume	0.03	0.03 (-0.03, 0.09)	0.73	-0.09 (-0.21, 0.04)	0.71	-0.001 (-0.08, 0.08)	0.98	-0.17 (-0.35, 0.02)	0.46
Total gray matter volume	-0.03	0.08 (-0.01, 0.16)	0.47	-0.02 (-0.21, 0.17)	0.920	0.05 (-0.07, 0.17)	0.73	-0.21 (-0.48, 0.06)	0.59
Normal white matter volume	0.07	-0.02 (-0.09, 0.05)	0.83	-0.16 (-0.31, -0.01)	0.35	-0.05 (-0.15, 0.05)	0.73	-0.12 (-0.33, 0.10)	0.73

© 2022 Hu YH et al. *JAMA Network Open.*

Abnormal white matter volume[e]		0.24		0.07 (-0.13, 0.27)	0.73		0.51 (0.09, 0.94)	0.32		0.10 (-0.17, 0.38)	0.73		0.82 (0.22, 1.43)	0.27
White matter fractional anisotropy		0.18		0.004 (-0.19, 0.20)	0.98		0.02 (-0.42, 0.46)	0.98		0.10 (-0.17, 0.38)	0.73		-0.13 (-0.75, 0.50)	0.83
Gray matter cerebral blood flow		0.54		0.19 (-0.00, 0.38)	0.37		0.44 (0.02, 0.86)	0.35		0.08 (-0.18, 0.34)	0.75		0.1 (-0.49, 0.70)	0.85

Data source: The Coronary Artery Risk Development in Young Adults (CARDIA), 1985-2016
CI=confidence interval.
a: q-value was calculated using Benjamini-Hochberg method for the adjustment of multiple comparison. q ≤0.1 was considered statistically significant.
b: For the volume of total brain, gray matter and hippocampus, n= 853; normal-looking whiter matter and abnormal white matter volume, n=846; white matter fractional anisotropy and mean diffusivity, n=777; and cerebral blood flow n=753.
c: All outcomes were standardized to Z-score values.
d: All models were adjusted for age, sex, race, education, field center, and Y25/Y30 measure. In Model 2, BMI, diabetes, physical activity level (log-transformed), current smoking status, and alcohol use were added. Model 3 was additionally adjusted for antihypertensive medication. Tissue volume measures models additionally adjusted for total intracranial volume; white matter fractional anisotropy and gray matter cerebral blood flow models additionally adjusted for total brain volume.
e: Abnormal white matter volume was log-transformed before standardizing to Z-score values.
f: Adjusted means based on the estimated model when intracranial volume/total brain volume and Y25/Y30 BP measure were standardized, and age and physical activity level centered.

© 2022 Hu YH et al. *JAMA Network Open.*

eTable 12. The association of diastolic blood pressure (DBP) trajectory with brain outcomes after adjustment of Y25/Y30 BP measure (reference DBP trajectory: Low-stable, overall n=853) [a,b,c,d]

	Low-stable (n=181)	Moderate-gradual (n=385)		Moderate-increasing (n=73)		Elevated-stable (n=181)		Elevated-increasing (n=33)	
	Mean[f]	β (95% CI)	*q*	β (95% CI)	*q*	β (95% CI)	*q*	β (95% CI)	*q*
**<u>Model 1 estimations</u>**									
Total brain volume	-0.05	-0.02 (-0.13, 0.09)	0.78	-0.02 (-0.09, 0.06)	0.75	0.01 (-0.06, 0.07)	0.85	-0.08 (-0.22, 0.05)	0.66
Total gray matter volume	-0.11	0.02 (-0.14, 0.17)	0.85	0.03 (-0.08, 0.14)	0.70	0.05 (-0.04, 0.14)	0.66	-0.11 (-0.30, 0.09)	0.66
Normal white matter volume	-0.01	-0.06 (-0.18, 0.07)	0.66	-0.07 (-0.15, 0.02)	0.61	-0.04 (-0.11, 0.03)	0.66	-0.05 (-0.21, 0.10)	0.70
Abnormal white matter volume[e]	0.50	-0.14 (-0.49, 0.22)	0.66	-0.22 (-0.47, 0.03)	0.60	-0.23 (-0.43, -0.03)	0.29	0.13 (-0.31, 0.58)	0.70
White matter fractional anisotropy	0.20	-0.21 (-0.57, 0.15)	0.66	-0.17 (-0.42, 0.08)	0.66	-0.05 (-0.26, 0.15)	0.70	-0.2 (-0.65, 0.25)	0.66
Gray matter cerebral blood flow	0.34	0.12 (-0.23, 0.46)	0.70	-0.04 (-0.28, 0.20)	0.79	0.06 (-0.13, 0.25)	0.70	-0.12 (-0.55, 0.31)	0.70
**<u>Model 2 estimations</u>**									
Total brain volume	0.02	-0.03 (-0.13, 0.08)	0.76	-0.02 (-0.10, 0.05)	0.70	-0.002 (-0.06, 0.06)	0.95	-0.09 (-0.23, 0.04)	0.67
Total gray matter volume	-0.04	0.01 (-0.15, 0.17)	0.94	0.02 (-0.09, 0.13)	0.83	0.04 (-0.05, 0.13)	0.67	-0.12 (-0.32, 0.08)	0.67
Normal white matter volume	0.07	-0.06 (-0.19, 0.06)	0.67	-0.07 (-0.16, 0.02)	0.67	-0.04 (-0.11, 0.03)	0.67	-0.05 (-0.21, 0.10)	0.67
Abnormal white matter volume[e]	0.52	-0.14 (-0.49, 0.22)	0.67	-0.22 (-0.47, 0.03)	0.67	-0.22 (-0.42, -0.02)	0.37	0.12 (-0.33, 0.56)	0.76
White matter fractional anisotropy	0.22	-0.23 (-0.58, 0.13)	0.67	-0.18 (-0.43, 0.07)	0.67	-0.07 (-0.27, 0.13)	0.67	-0.2 (-0.65, 0.24)	0.67
Gray matter cerebral blood flow	0.57	0.12 (-0.23, 0.47)	0.67	-0.03 (-0.27, 0.21)	0.85	0.07 (-0.13, 0.26)	0.67	-0.15 (-0.58, 0.28)	0.67
**<u>Model 3 estimations</u>**									
Total brain volume	0.02	-0.005 (-0.12, 0.11)	0.96	-0.01 (-0.09, 0.07)	0.96	0.004 (-0.06, 0.07)	0.96	-0.06 (-0.21, 0.08)	0.96
Total gray matter volume	-0.04	0.03 (-0.13, 0.20)	0.96	0.04 (-0.08, 0.16)	0.96	0.05 (-0.04, 0.14)	0.96	-0.09 (-0.29, 0.12)	0.96
Normal white matter volume	0.07	-0.04 (-0.17, 0.09)	0.96	-0.05 (-0.14, 0.04)	0.96	-0.04 (-0.11, 0.03)	0.96	-0.02 (-0.19, 0.14)	0.96

© 2022 Hu YH et al. *JAMA Network Open.*

Abnormal white matter volume[e]		0.51		-0.24 (-0.61, 0.13)	0.96		-0.31 (-0.57, -0.04)	0.41		-0.25 (-0.45, -0.05)	0.41		-0.03 (-0.50, 0.43)	0.96
White matter fractional anisotropy		0.23		-0.14 (-0.51, 0.23)	0.96		-0.10 (-0.37, 0.16)	0.96		-0.05 (-0.25, 0.15)	0.96		-0.08 (-0.54, 0.39)	0.96
Gray matter cerebral blood flow		0.57		0.13 (-0.23, 0.48)	0.96		-0.03 (-0.28, 0.23)	0.96		0.07 (-0.13, 0.26)	0.96		-0.15 (-0.60, 0.30)	0.96

Data source: The Coronary Artery Risk Development in Young Adults (CARDIA), 1985-2016

CI=confidence interval.

a: q-value was calculated using Benjamini-Hochberg method for the adjustment of multiple comparison. q ≤0.1 was considered statistically significant.

b: For the volume of total brain, gray matter and hippocampus, n= 853; normal-looking whiter matter and abnormal white matter volume, n=846; white matter fractional anisotropy and mean diffusivity, n=777; and cerebral blood flow n=753.

c: All outcomes were standardized to Z-score values.

d: All models were adjusted for age, sex, race, education, field center, and Y25/Y30 measure. In Model 2, BMI, diabetes, physical activity level (log-transformed), current smoking status, and alcohol use were added. Model 3 was additionally adjusted for antihypertensive medication. Tissue volume measures models additionally adjusted for total intracranial volume; white matter fractional anisotropy and gray matter cerebral blood flow models additionally adjusted for total brain volume.

e: Abnormal white matter volume was log-transformed before standardizing to Z-score values.

f: Adjusted means based on the estimated model when intracranial volume/total brain volume and Y25/Y30 BP measure were standardized, and age and physical activity level centered.

© 2022 Hu YH et al. *JAMA Network Open.*

eTable 13. The association of mean arterial pressure (MAP) trajectory with brain outcomes after adjustment of Y25/Y30 BP measure (reference MAP trajectory: Low-stable, overall n=853) [a,b,c,d]

	Low-stable (n=181)	Moderate-gradual (n=385)		Moderate-increasing (n=73)		Elevated-stable (n=181)		Elevated-increasing (n=33)	
	Mean[f]	β (95% CI)	*q*	β (95% CI)	*q*	β (95% CI)	*q*	β (95% CI)	*q*
**Model 1 estimations**									
Total brain volume	-0.06	0.01 (-0.05, 0.07)	0.87	0.03 (-0.09, 0.14)	0.86	-0.01 (-0.09, 0.07)	0.89	-0.08 (-0.22, 0.06)	0.60
Total gray matter volume	-0.11	0.05 (-0.04, 0.14)	0.60	0.06 (-0.11, 0.23)	0.69	0.05 (-0.07, 0.16)	0.67	-0.09 (-0.29, 0.12)	0.67
Normal white matter volume	-0.01	-0.029 (-0.10, 0.04)	0.67	-0.04 (-0.17, 0.10)	0.81	-0.07 (-0.16, 0.01)	0.35	-0.07 (-0.23, 0.09)	0.67
Abnormal white matter volume[e]	0.34	-0.05 (-0.26, 0.15)	0.81	0.32 (-0.06, 0.69)	0.35	-0.02 (-0.27, 0.23)	0.91	0.36 (-0.09, 0.81)	0.35
White matter fractional anisotropy	0.17	-0.03 (-0.23, 0.18)	0.89	-0.19 (-0.57, 0.19)	0.64	-0.21 (-0.47, 0.04)	0.35	-0.18 (-0.64, 0.28)	0.67
Gray matter cerebral blood flow	0.29	0.14 (-0.05, 0.34)	0.37	0.31 (-0.06, 0.69)	0.35	-0.02 (-0.27, 0.23)	0.91	-0.08 (-0.51, 0.36)	0.87
**Model 2 estimations**									
Total brain volume	0.01	0.01 (-0.06, 0.07)	0.87	0.03 (-0.08, 0.15)	0.79	-0.01 (-0.09, 0.07)	0.84	-0.08 (-0.22, 0.06)	0.64
Total gray matter volume	-0.05	0.04 (-0.05, 0.14)	0.64	0.08 (-0.09, 0.25)	0.64	0.04 (-0.07, 0.16)	0.72	-0.10 (-0.30, 0.11)	0.64
Normal white matter volume	0.06	-0.032 (-0.10, 0.04)	0.65	-0.03 (-0.17, 0.10)	0.82	-0.07 (-0.17, 0.02)	0.45	-0.07 (-0.22, 0.09)	0.69
Abnormal white matter volume[e]	0.36	-0.05 (-0.25, 0.16)	0.82	0.31 (-0.07, 0.69)	0.45	-0.007 (-0.26, 0.25)	0.96	0.34 (-0.11, 0.80)	0.45
White matter fractional anisotropy	0.17	-0.03 (-0.23, 0.17)	0.84	-0.19 (-0.56, 0.19)	0.64	-0.22 (-0.48, 0.04)	0.45	-0.16 (-0.62, 0.30)	0.76
Gray matter cerebral blood flow	0.52	0.17 (-0.03, 0.37)	0.45	0.33 (-0.04, 0.70)	0.45	0.02 (-0.23, 0.27)	0.90	-0.10 (-0.53, 0.33)	0.82
**Model 3 estimations**									
Total brain volume	0.01	0.02 (-0.05, 0.08)	0.95	0.07 (-0.05, 0.19)	0.95	0.01 (-0.07, 0.10)	0.95	-0.03 (-0.18, 0.12)	0.95
Total gray matter volume	-0.05	0.06 (-0.03, 0.15)	0.95	0.12 (-0.05, 0.30)	0.95	0.08 (-0.05, 0.20)	0.95	-0.04 (-0.25, 0.18)	0.95
Normal white matter volume	0.06	-0.03 (-0.10, 0.05)	0.95	-0.01 (-0.15, 0.13)	0.97	-0.06 (-0.15, 0.04)	0.95	-0.03 (-0.20, 0.14)	0.95

© 2022 Hu YH et al. *JAMA Network Open.*

Abnormal white matter volume[e]		0.36		-0.08 (-0.29, 0.13)	0.95		0.21 (-0.19, 0.60)	0.95		-0.09 (-0.36, 0.19)	0.95		0.20 (-0.29, 0.68)	0.95
White matter fractional anisotropy		0.17		0.004 (-0.20, 0.21)	0.97		-0.08 (-0.48, 0.31)	0.95		-0.14 (-0.41, 0.13)	0.95		-0.01 (-0.50, 0.48)	0.97
Gray matter cerebral blood flow		0.52		0.17 (-0.03, 0.38)	0.95		0.34 (-0.05, 0.73)	0.95		0.03 (-0.23, 0.30)	0.97		-0.07 (-0.54, 0.39)	0.95

Data source: The Coronary Artery Risk Development in Young Adults (CARDIA), 1985-2016
CI=confidence interval.
a: q-value was calculated using Benjamini-Hochberg method for the adjustment of multiple comparison. $q \leq 0.1$ was considered statistically significant.
b: For the volume of total brain, gray matter and hippocampus, n= 853; normal-looking whiter matter and abnormal white matter volume, n=846; white matter fractional anisotropy and mean diffusivity, n=777; and cerebral blood flow n=753.
c: All outcomes were standardized to Z-score values.
d: All models were adjusted for age, sex, race, education, field center, and Y25/Y30 BP measure. In Model 2, BMI, diabetes, physical activity level (log-transformed), current smoking status, and alcohol use were added. Model 3 was additionally adjusted for antihypertensive medication. Tissue volume measures models additionally adjusted for total intracranial volume; white matter fractional anisotropy and gray matter cerebral blood flow models additionally adjusted for total brain volume.
e: Abnormal white matter volume was log-transformed before standardizing to Z-score values.
f: Adjusted means based on the estimated model when intracranial volume/total brain volume and Y25/Y30 BP measure were standardized, and age and physical activity level centered.

eReferences

1. Launer LJ, Lewis CE, Schreiner PJ, et al. Vascular factors and multiple measures of early brain health: CARDIA brain MRI study. *PloS one*. 2015;10(3)

2. Allen NB, Siddique J, Wilkins JT, et al. Blood pressure trajectories in early adulthood and subclinical atherosclerosis in middle age. JAMA. 2014;311(5):490-497.

3. Nagin DS, NAGIN D. Group-based modeling of development. Harvard University Press; 2005.

© 2022 Hu YH et al. *JAMA Network Open.*
